# The Challenges and Limitations of Validating Satellite-Derived Datasets Using Independent Measurements: Lessons Learned from Essential Climate Variables

**DOI:** 10.1007/s10712-025-09898-4

**Published:** 2025-08-11

**Authors:** Mary Langsdale, Tijl Verhoelst, Adam Povey, Nick Schutgens, Thomas Dowling, Jean-Christopher Lambert, Steven Compernolle, Stefan Kern

**Affiliations:** 1 Department of Geography, King’s College London, Bush House NE Wing, London, WC2B 4BG UK; 2 National Centre for Earth Observation, King’s College London, Bush House NE Wing, London, WC2B 4BG UK; 3 Atmospheric Data Synergies, Royal Belgian Institute for Space Aeronomy (BIRA-IASB), Ringlaan 3, 1180 Brussels, Belgium; 4 National Centre for Earth Observation, University of Leicester, 92 Corporation Road, Leicester, LE4 5SP UK; 5 Department of Earth Science, Vrije Universiteit Amsterdam, De Boelelaan 1100, 1081 HZ Amsterdam, The Netherlands; 6 School of Environment and Space Institute, University of Auckland, 23 Symonds Street, 1142 Auckland, New Zealand; 7 Integrated Climate Data Center – Earth and Societies Research Hub, University of Hamburg, Grindelberg, 20144 Hamburg, Germany

**Keywords:** Satellite validation, Cal/Val, In-situ measurements, Uncertainty analysis, Essential climate variable

## Abstract

Validation of satellite-derived essential climate variable (ECV) datasets requires comparison against independent measurements. These independent measurements, which include ground-based, airborne, and other non-satellite-based measurements, are typically the product of a different measurement system and may include some contribution from models. These reference data therefore have their own characteristics, uncertainties, and limitations which must be accounted for in the validation process. In addition, they typically differ from the data to be validated in spatio-temporal resolution, sensitivity, and sampling. As such, comparisons to independent data do not necessarily yield clear feedback on the quality of satellite data and insufficient awareness of these issues can lead to erroneous interpretation. This is the cost of leaving the laboratory and studying the real world. In this review paper, we examine the challenges and limitations of evaluating satellite-derived datasets with independent measurements, using examples across different ECVs within the terrestrial, ocean, and atmospheric domains. Drawing from other studies, we discuss issues with the reference datasets themselves, issues specific to use of these data for validation, and issues resulting from the comparison methodology. We conclude with recommendations to the community based on this review. In this, we highlight the importance of continued efforts towards (1) advancing uncertainty modelling of reference datasets and quality control knowledge and procedures, (2) establishing and communicating limitations in reference data, (3) reference data (and metadata) timeliness and preservation, and (4) best practices for the validation methodologies that address the spatio-temporal differences of the measurements.

**Article Highlights**

- First review of the challenges and limitations of validating satellite-derived datasets across different scientific communities
- Challenges and limitations considered across all stages of the validation process
- Comprehensive recommendations to the community provided based on the review

## Introduction

The Global Climate Observing System (GCOS) defines an essential climate variable (ECV) as a singular or collective set of well-defined physical, chemical, or biological quantities that critically contribute to the characterization of the Earth’s climate. The GCOS Implementation Plan (IP) (GCOS 2022) currently specifies fifty-five different ECVs, although they are in the process of rationalizing this by combining existing ECVs. Most ECVs are observed operationally by satellite instruments from which we retrieve climate data records (CDRs) (also called Earth System Data Records, ESDRs).

To enable all satellite data users to readily evaluate the fitness for purpose of the data, the Quality Assurance Framework for Earth Observation (QA4EO) requires that every satellite-derived product includes a documented and fully traceable indicator of the data quality. As defined in Loew et al. (2017), this quality assessment requires uncertainty analysis of and traceability on the product(s). More concretely, the GCOS IP defines quantifiable requirements on the ECV data records in terms of resolution, timeliness, measurement uncertainty and stability. While verification of the true resolution is sometimes part of validation studies (e.g., Keppens et al. 2015, for the vertical resolution in atmospheric profile retrievals), we focus here specifically on the quantification and verification of the uncertainty and stability.

For a satellite-derived product, there are generally two approaches to this. The first is based on the metrological analysis of the measurement process, creating a traceability chain or diagram that results in prognostic or ex-ante uncertainty estimates (Merchant et al. 2017; Popp and Mittaz 2022). The second is based on the comparison of the data to other dataset(s) of (ostensibly) the same measurand (a quantity to be measured) to produce diagnostic or ex-post-quality indicators (Verhoelst et al. 2025). The approaches for each have their unique challenges, but in this paper we consider the latter, which the scientific community calls evaluation or validation. In this context, evaluation is the generic process of judging the quality of the product, whereby validation is defined by the Committee on Earth Observation Satellites (CEOS) as assessing the quality of the data by means independent of its creation (Loew et al. 2017). Our review will focus on validation specifically.

The validation process aims to quantify the extent to which a satellite-derived data product represents the ‘truth’, from which the user can make a judgement as to the quality of the measurement and whether it is fit for their purpose. This validation process can typically provide information about the bias, precision, long-term stability, patterns, dependence on ancillary parameters, and other quality information that can be communicated to data users. For all variables, including ECVs, the results of these validation exercises form the basis of the ongoing quality evaluation of the products and they point to areas for further product development. The credibility and reliability of the validation depends on the methodology used as well as the fitness-for-purpose, accuracy, and reliability of the reference datasets used. As Povey and Grainger (2015) remark, a good validation requires at the very least that the conclusions would not change were an additional reference dataset introduced.

As part of the push for rigorous quality evaluation of ECV products in recent years, the concept of a Fiducial Reference Measurement (FRM) has been developed (Zibordi et al. 2014; Goryl et al. 2023). The CEOS Working Group on Calibration and Validation (WGCV) defines an FRM as an independent, fully characterized, and traceable measurement, tailored specifically to the calibration/validation needs of satellite-based instruments making measurements of a particular measurand, that follow the guidelines framework outlined by the QA4EO. Essential in this FRM concept is a standardized and traceable uncertainty characterization of the reference measurements along all steps of the traceability chain from SI standards to final reported value(s) for the measurand. Fundamentally, the FRM framework provides guidelines towards user and producer confidence in the validation of satellite-derived products.

The maturity of any reference measurement can be assessed using the CEOS-FRM maturity assessment framework, which specifies the level (or stage) to which a dataset adheres to those standards. These standards can be broadly split into three categories: (1) the FRM compliance of the reference measurement independent from the validation process, (2) the FRM compliance of the reference dataset when used for validation of a given class of sounders, and (3) the FRM compliance of the comparison methodology for the validation process and its output.

The CEOS-FRM maturity assessment framework links with an increasing push for assessment tools such as maturity matrices (e.g., Bates and Privette 2012) which draw upon the established technology or technical readiness level (TRL) framework to assess the status of climate data records (CDRs). An example of a maturity matrix applied to CDRs is shown in Su et al. (2018). The maturity matrix framework has also been extensively used in the Copernicus Climate Change Service (C3S)’s Evaluation and Quality Control (EQC) function, which assesses product service and readiness (Lacagnina et al. 2022). Other examples include the Earthnet Data Assessment Project (EDAP), who have developed a maturity matrix approach to characterize satellite data completeness (EDAP 2022), and the Gap Analysis for Integrated Atmospheric ECV Climate Monitoring (GAIA-CLIM) maturity matrix (Thorne et al. 2015), which has been applied as a standard to several ground-based monitoring networks for atmospheric variables (e.g., GRUAN and NDACC).

One should distinguish between (i) the maturity/completeness of the satellite product, (ii) that of the reference networks (Goryl et al. 2023), both already mentioned above, and (iii) validation maturity/completeness.

However, it is recognized by key stakeholders (such as the space agencies and ground-based monitoring programmes) that despite the push towards FRM and maturity assessments, the uncertainty budgets currently reported with reference datasets are often incomplete. Specifically, many do not cover all underlying sources of uncertainty, nor do they differentiate (sufficiently) between random and systematic error sources. Furthermore, little is known about the structure or magnitude of correlations in space, time, or other dimensions in most error sources.

In an attempt to rectify this, the key data providers maintain a long-term discussion on validation needs, gaps, and capacities. Several dedicated initiatives have been set up, such as ESA’s FRM4XXX projects (e.g., Goryl et al. 2023) with (CEOS) FRM maturity application examples for FRM4GHG (Sha et al. 2024) and FRM4DOAS (Van Roozendael et al. 2024), CEOS WGCV Best Practice Validation Protocols (e.g., Duncanson et al. 2020), and European Organisation for the Exploitation of Meteorological Satellites (EUMETSAT) CO2M Cal/Val studies. In this regard, they focus not only on a standardized uncertainty characterization but also on timeliness of data provision, harmonized processing, and formatting across the network(s). These initiatives have been recognized and appreciated by many within the cal/val community and efforts continue to elevate reference datasets to FRM status. However, there are still limitations and challenges that prevent this from being achieved across all ECV validation studies.

The general aims of this review paper are to raise awareness of these limitations and challenges by reviewing relevant studies and to present recommendations to users and producers of these products to manage or mitigate these themes and issues.

To achieve these objectives, we start in Sect. 2 with a review of the limitations and challenges of the individual measurements themselves, considering issues of traceability, accessibility, and uncertainty characterization. In Sect. 3, challenges and limitations of using these datasets for specific validation activities are then considered. These include measurand differences, independence issues, validation capacity and completeness, and representative sampling. In Sect. 4, the review is extended to consideration of the validation and comparison methodology, considering harmonization of reference and satellite datasets, construction of uncertainty budgets, and co-location/sensitivity mismatches. Sections 2, 3, 4 also include an overview of potential solutions to the challenges discussed therein, drawing on examples from studies that have deployed such techniques. Finally, in Sect. 5, we conclude with recommendations to the community based on our discussion of these challenges and the potential solutions identified. Throughout, examples will be considered from different ECVs within terrestrial, ocean, and atmospheric domains.

For definitions of terms commonly used in Earth Observation (EO) validation (such as accuracy, measurement error, measurement uncertainty), we utilize the terminology defined in Table 1 of Loew et al. (2017).

## Limitations of the Reference Measurements Themselves

As discussed in Sect. 1, reference measurements will typically have limitations and challenges even before they are used in validation studies. Considering the CEOS-FRM maturity assessment framework, these include:

1. The traceability of the reference measurement to a community-agreed standard,
2. The accessibility of the reference measurement and associated metadata, including the availability of documented protocols for the measurement process and data quality management, and
3. The availability and adequacy of a complete uncertainty budget.

In this section, limitations and challenges specific to each of these issues are reviewed.

### Traceability

As detailed in the International Vocabulary of Metrology (BIPM et al. 2012), traceability is a property of a measurement whereby the result can be related to a reference or standard through a documented, unbroken chain of calibrations (each contributing to the uncertainty). In theory, this means that the instruments used to collect reference measurements must undergo regular calibration against a traceable standard (preferably SI). In practice, this is not always achievable. SI-traceable standards may not be available in such circumstances (particularly for observations from orbit), calibration facilities may be unavailable, or instrumentation may be non-recoverable post-campaign. The traceability of satellite observations will also vary according to the mission, with better on-board calibration techniques on missions with more strict mission requirements on uncertainty and stability.

To illustrate this, consider surface temperature reference measurements, which are typically made using ground-based infrared radiometers (Guillevic et al. 2018). These can be calibrated against SI-traceable blackbody calibration sources. However, to derive surface temperatures from the infrared brightness temperatures recorded by the radiometers, the user must also have information on the surface emissivity, a parameter which describes the amount of radiation emitted at a particular wavelength by that surface (Li et al. 2013). Various methods of measuring emissivity for validation studies have been developed (summarized in Langsdale et al. 2020), with laboratory methods recommended for use (Guillevic et al. 2018). However, there are no SI (or other community-agreed) traceable standards against which to relate the performance of these methods. Furthermore, there are limited facilities available to evaluate any components which can be calibrated (Langsdale et al. 2021). The only option for quality assessment of these emissivity data is inter-comparison studies, but such exercises between ostensibly equivalent laboratory setups have shown discrepancies often overlooked by land surface temperature (LST) validation studies (Langsdale et al. 2021). LST retrieval algorithms are sensitive to errors in emissivity (Jiménez-Muñoz and Sobrino 2006), such that the discrepancies in emissivity result in differences in calculated LSTs that exceed the GCOS requirements for LST accuracy, even before considering contributions from other known (and considerable) sources of uncertainty (Langsdale et al. 2021).

This specific example questions whether such laboratory emissivity setups are fit-for-purpose in LST validation exercises. More broadly, this example underscores a common limitation of reference datasets across various variables: data can be unreliable in a manner that is difficult to detect when parameters of the retrieval are not anchored to an established standard that provides traceability beyond the specific setting of that observation. Further research is required to develop robust traceability frameworks for measurements used in the derivation of ECV reference observations, notably those which the ECV are highly sensitive to.

### Accessibility

There are two key challenges regarding the accessibility of reference measurements: metadata and data provision.

#### Metadata

Metadata provides essential context and information about datasets that enable the user to understand, interpret, and use datasets appropriately. The metadata of a dataset includes both global metadata (high-level information applicable to an entire dataset, e.g., collector or processing methodology) and observational or local metadata (specific to individual observations or data points within a dataset, e.g., timestamp, location). The importance of metadata within the context of validation datasets is recognized by Goryl et al. (2023), who highlight good metadata, documentation, and reporting as a defining and mandatory characteristic of an FRM. Unfortunately, many datasets used in validation activities do not have sufficient or sufficiently accessible metadata to enable users to understand precisely what the data express. This can result in datasets being incorrectly used, with potential for misunderstandings to propagate downstream.

Consider again the example of emissivity discussed in Sect. 2.1. In studies where scientists do not have access to field or laboratory methods of emissivity measurement, many instead use data from spectral libraries, which are public datasets of emissivity spectra. The most cited of these is the ECOSTRESS (formerly ASTER) spectral library (Meerdink et al. 2019; Baldridge et al. 2009). Data from this library have been used in several validation studies for LST, surface albedo, aerosol, and other ECVs (Michel et al. 2021; Yamamoto and Irie 2023; Sánchez-Zapero et al. 2023; Niclòs et al. 2021; Ru et al. 2023; Ghent et al. 2024).

**Table 1:** Metadata for the ‘conifer’ spectrum, taken from the ECOSTRESS spectral library (Meerdink et al. 2019)

Name	Conifer
Type	Vegetation
Class	Tree
Genus	Unknown
Species	Unknown
Sample No	Conifer
Owner	JHU
Wavelength Range	All
Origin	Spectral data from measurements by Barry Rock (VNIR-SWIR) and Jack Salisbury (MWIR-TIR); see file vegetata.doc for details
Collection Date	Unknown
Description	Conifer trees. Spectra were assembled from two segments; the bidirectional VNIR and SWIR comprising segment one, and the hemispherical MWIR and TIR comprising segment two. Both segments used needle piles to simulate a conifer canopy
Measurement	Bidirectional and directional hemispherical reflectance
First Column	X
Second Column	Y
X Units	Wavelength (micrometres)
Y Units	Reflectance (per cent)
First X Value	0.302
Last X Value	14
Number of X Values	550
Additional Information	None

Filename: “vegetation.tree.unknown.unknown.all.conifer.jhu.becknic.spectrum.txt”

The contents of this library are openly available. Each file contains both metadata and data with additional reporting available in Baldridge et al. (2009) and Meerdink et al. (2019). Not only do these files and the accompanying reports lack uncertainty values or an assessment of measurement quality, but there can be limited information about the equipment and methods used, invalid cross-referencing, and inconsistent levels of detail provided from sample to sample. For example, Table 1 shows the metadata for a conifer spectrum from the ECOSTRESS spectral library, which has been used in assessments of operational LST and emissivity retrieval algorithms (Hulley et al. 2021). Neither the metadata for this file nor the accompanying studies contain any information about a number of factors which could influence the derived emissivity, including the type of conifer, the condition of the needles during measurement, sample preparation techniques, and post-processing methodology. There is a reference to a document (‘vegetata.doc’) which is not available within the database. Finally, there are no images available of the sample which would help a user to determine whether the spectra are representative of the claimed surface and, therefore, suitable for their particular study. A user could easily mistakenly select this spectra, assuming it to be representative of their study site when, in reality, it considered a different material with a similar name. Complete metadata, or at the very least including imagery, could avoid these problems. Creating metadata is easiest to do upon data generation but can be very difficult to determine retrospectively.

#### Data Provision

Ensuring the traceability and accessibility of reference datasets is often hindered by issues related to data ownership, proprietary restrictions, and global data repositories.

Historic measurements pose unique challenges for accessibility and long-term preservation. Many historic datasets are stored in formats that may no longer be compatible with modern data systems or software tools. The rapid evolution of digital storage technologies and data formats can render these datasets difficult to access, read, or analyse without significant effort to reformat or convert the data. In some cases, physical data media (such as tapes or early disk drives) may have degraded, potentially leading to data loss or corruption. In others cases, such as early sea surface temperature (SST) measurements, observations were mostly noted down by hand (e.g., in ship logbooks or diaries), are rarely digitized, and might not be accessible. Furthermore, there may be limited documentation accompanying historic datasets, regarding measurement equipment and standards, or there may be researcher mistakes and/or subjectivity, as discussed in depth in the DOCU-CLIM project (Burgdorf et al. 2023).

This problem is exacerbated by the lack of centralized data repositories and the decentralized nature of archival efforts, which can result in data being stored across several institutions or geographic locations, further complicating the process of retrieving and sharing historic reference datasets.

Despite these factors, it is important that historical datasets are preserved and disseminated. Historic measurements play a critical role in long-term stability assessments of CDRs, with SST measurements, for example, providing one of the longest instrumental records of the Earth’s climate which is crucial information on climate stability and satellite performance. Preservation of historical datasets is particularly relevant when current retrieval schemes are applied to historical satellite data to extend ECV data records to times before the earliest reference measurements. This is the case for several atmospheric products of ECV precursors (e.g., the tropospheric column of formaldehyde).

Data provision is also an issue for recently collected ground-based datasets. While satellite data are often made available for analysis shortly after collection, many reference datasets undergo a lengthy post-processing period before being made publicly available. Such delays reduce the ability to conduct real-time or near-real-time validation, which is increasingly important for timely calibration and validation of satellite sensors. There is a real value to having reference data accessible soon after collection, even if only in a preliminary format.

### Incomplete Uncertainty Characterization

The ability to usefully characterize the associated uncertainties varies substantially between different ECVs. However, reference data which has generic or absent uncertainty information can still be useful, not least because it may be the only reference data available.

For example, many historic measurements do not have complete uncertainty characterization. This is partly due to increased emphasis on uncertainty characterization in recent years, as evidenced by projects such as ESA, EUMETSAT, and Copernicus activities on FRMs (Goryl et al. 2023; Le Menn et al. 2019). However, retrospective uncertainty characterization is also challenging as the user often lacks the necessary information to compute the uncertainty. For example, calibration procedures for earlier instruments may not be well-documented which may lead to incorrect assumptions in the derivation of uncertainties or gaps in uncertainty calculation. Despite the lack of complete uncertainty characterization, historic datasets can be highly valuable for extending the spatio-temporal capacity of validation studies and, therefore, they are considered valuable.

Evaluation of sea ice ECV quantities, such as sea ice thickness, is a relevant example of the issues discussed above, particularly when evaluating time series that extend before 2000. The available observations often lack full uncertainty characterization. Examples include sea ice draft obtained by upward looking sonar (ULS) from submarines (Kwok and Rothrock 2009; Rothrock and Wensnahan 2007). While valuable, full uncertainty characterization is difficult because these data are often poorly documented, with limited information about the exact location and time of the observation, and insufficient information about the instruments’ properties.

Such problems are not unique to historic datasets. In remote regions, such as the poles, the only reference data available may not comply with FRM standards due to the logistics of working in harsh or remote environments. We highlight the value of manual ship-based observations of sea ice parameters. Particularly in the Southern Ocean, which is notoriously undersampled, such observations can play a major role and have been efficiently used for evaluation despite lacking full uncertainty characterization (e.g., Kern et al. 2019; Fons et al. 2023).

Human mistakes are another cause of incomplete uncertainty characterization. Examples of this could be coding bugs, writing the wrong number in the field log, or using the wrong sensor calibration. In the best case scenario, the user is aware of the mistake and can attempt to estimate the resulting uncertainty or remove the erroneous data. In the worst case, the user faces ‘unknown unknowns’ (Povey and Grainger 2015) where an improper use of reference data may result in mischaracterization of the ECV quality. In these situations it can be near impossible to estimate the resultant uncertainty.

It is important to emphasize the potential for reverse feedback from satellite validation to reference datasets. While reference data are in principle “the truth", satellite validation often yields valuable feedback on the quality of these reference data. A satellite dataset can serve as a transfer standard to assess the homogeneity of ground-based network data, allowing identification of station or time-series anomalies. With the ever-increasing quality of satellite remote sensing data, the potential to identify issues in the reference data grows. This “reverse feedback" mechanism was demonstrated by Fioletov et al. (2008), who used an ensemble of validated satellite total ozone column datasets to detect issues with the ground-based Dobson and Brewer instruments typically used as a reference in such work. The outcome of such studies was helpful in shaping later field campaigns, leading to significant improvements in the Dobson and Brewer data QA/QC, their calibration, and in the characterization of uncertainties. Even today, comparisons to Sentinel-5P TROPOMI ozone column measurements reveal quality issues in the state-of-the-art EUBREWNET Brewer measurements (A. Redondas, priv. comm.). This approach has also been applied to aerosol optical depth (AOD) and SST products (Schutgens et al. 2020; Kennedy et al. 2012).

Finally, we also must acknowledge that our understanding of the physical Earth System is developing and with it our awareness of limitations within validation datasets. For example, oceanic rogue waves have only recently been identified within ocean wave validation datasets (Breunung and Balachandran 2024), and then observed at scale (Häfner et al. 2018) with the aid of machine learning and satellite observations (Teutsch 2022; Teutsch et al. 2020). Before the advent of the technologies permitted the unambiguous identification of rogue waves, they were often referred to as ‘maritime folklore’ (Kharif and Pelinovsky 2003). Members of the validation community must be careful to not assume that a validation dataset, particularly when derived from sparsely distributed point measurements, captures all of the system dynamics of complex natural phenomena. This example also highlights the potential for machine learning and artificial intelligence (AI) techniques to discover physical processes (and provide new context to existing data), which is anticipated to continue as AI is used more widely within the scientific community.

### Potential Solutions

As satellite validation depends heavily on high-quality reference measurements and historical datasets, it is essential to address the limitations of such data discussed above to facilitate the continued improvement of ECVs.

#### Identification of Limitations

As a first step, there is an urgent need for systematic identification of limitations within different datasets, such as assessing the extent to which (full) uncertainty characterization has been conducted or evaluating the adequacy and fitness-for-purpose of accompanying documentation. Leveraging existing maturity assessment frameworks, this process should involve both quantitative approaches (e.g., inter-comparison studies) and qualitative methods (e.g., evaluating accompanying metadata to understand the traceability of variables).

An exemplary means of achieving this evaluation (and communicating it) would be the production of an uncertainty tree. An uncertainty tree is a visual tool from the metrology community used to systematically break down and assess sources of uncertainty in a data product. In Earth Observation, this can be used to understand how uncertainties in raw observations propagate through processing chains to final outputs. Typically, an uncertainty tree applied to Earth Observation products would include instrumental uncertainty (for example, sensor noise and calibration errors), algorithm uncertainty (for example, model assumptions or simplifications) and aggregation uncertainty (for example, sampling or interpolation errors). Examples of uncertainty trees for satellite-derived and reference data products can be found in Merchant et al. (2017); Mittaz et al. (2019); Popp and Mittaz (2022); Ma et al. (2020) and Wimmer and Robinson (2016).

Uncertainty trees can also be a helpful tool to identify input parameters without well-characterized or understood uncertainties, such as the example of emissivity within this section. Equally important is the need for data providers to be honest about the limitations within their datasets. Rather than omitting information about missing inputs, providers should explicitly state, for example, when uncertainty budgets lack specific components.

Finally, we advocate for the continuation and enhancement of activities aimed at assessing and improving the maturity of reference datasets, such as ESA and EUMETSAT’s FRM4XXX projects and the CEOS-FRM maturity assessment framework. Together, this process can be used to identify areas for improvement as well as areas of increased confidence.

#### Addressing Limitations

Following maturity assessment, steps should be taken to rectify limitations where possible. Considering first the uncertainty characterization, there are several frameworks available to support scientists with this, which can broadly be grouped into two categories:

1. The construction of detailed traceability chains and uncertainty propagation and/or sensitivity studies on underlying assumptions (for example, Sha et al. 2024; Mittaz et al. 2019; Kern et al. 2019).
2. Inter-comparison campaigns and exercises

Regarding the latter point, an example of this could be to organize for (large) sets of reference instruments brought together at a single site to obtain co-located measurements allowing a direct comparison and thus assessment of their mutual consistency. For instance, in June 2024 more than 40 MAX-DOAS instruments and complementary systems by more than 30 groups worldwide were brought together in the Third Cabauw Intercomparison of Nitrogen Dioxide Measuring Instruments (CINDI-3) campaign organized under the joint umbrella of the ACTRIS Research Infrastructure, ESA and the Network for the Detection of Atmospheric Composition Changes (NDACC).

We encourage both routine and independent inter-comparisons between datasets. Routine comparisons by independent groups reduce the likelihood that simple misunderstandings or mistakes propagate beyond the data collectors. Evaluation by researchers uninvolved in data production provides scrutiny of assumptions in the validation process that data producers may share for practical reasons; this has been the practice within the ESA-CCI programme (e.g., Kern et al. 2019, 2022). Comparison of all datasets can identify unacknowledged or unnoticed sources of error, particularly where those error sources result from differences of intention between communities with different goals and aspirations.

In cases where there is incomplete uncertainty characterization, where possible it is highly important that one estimate it, in the full knowledge that your estimate will be imperfect but is much better than ignorance.

#### Accessibility and Data Management

To promote accessibility, data providers should strive to include comprehensive and accurate metadata and documentation within their datasets to enable responsible data use. Suitable examples of documentation can be found in the FRM frameworks (for example, CEOS-WGCV 2023). Fundamentally, this involves ensuring that users understand what was measured and how it was measured, enabling them to evaluate the dataset’s suitability for their specific study. This requirement is especially pertinent given the growing adoption of open-access data standards. The FAIR (Findability, Accessibility, Interoperability, and Reusability) Guiding Principles are a notable example of how to practically address and improve data management (Wilkinson et al. 2016).

Such rigorous attention to detail must extend across all datasets, including those from novel or experimental techniques. While we highlight the importance of FRM-compliant reference data, we emphasize additionally the value of non-FRM datasets. These datasets can play a crucial role in capturing situations that are challenging to measure using FRM standards. In our view, any field measurement conducted to enhance understanding of small-scale variability as a function of environmental conditions can be valuable, even if not FRM-compliant, provided that it is accompanied by an adequate set of metadata.

There have been a number of impressive data rescue initiatives in recent years. Examples include the global documentary climate dataset for climate reconstructions (DOCU-CLIM) (Burgdorf et al. 2023), the international Atmospheric Circulation Reconstructions over the Earth (ACRE) (Allan et al. 2016), and the World Meteorological Organization’s MEditerranean climate DAta REscue (MEDARE) initiative. Preservation attempts must seek to address biases due to different measurement methods, as demonstrated by the Met Office’s work with SST measurements (Kennedy et al. 2011), and those introduced by researcher subjectivity. Such challenges highlight the need for collaboration between physical scientists, historians, and social scientists to construct robust, long-term climate records.

Closely related to these preservation efforts is the recognition that numerous existing datasets may be at risk of data loss. This underscores the necessity of establishing a dedicated data portal for “homeless" reference data. Valuable insights can be drawn from the atmospheric science community, which has developed resources such as the ‘Homeless’ data portal (ATMO-ACCESS 2023) and contributed to ESA’s Earth Observation Validation Data Centre (EVDC) (https://evdc.esa.int/). Both portals serve as exemplary models, featuring well-defined standardized protocols for uploading data, including comprehensive metadata and documentation.

## Limitations of the Reference Datasets for Specific Validation Activities

To be considered FRM-compliant, a reference dataset must meet further standards within the CEOS-FRM maturity assessment framework. These include:

- the independence of the reference dataset from the data to be validated,
- the representativeness of the reference dataset to the satellite data characteristics, and
- the utility of the reference dataset for a class of satellite data.

This section explores limitations of reference datasets to meet these standards, addressing measurand differences, independence issues, validation capacity and completeness, and representativeness.

### Measurand Differences

Within the metrology community, the issues discussed in this section might be referred to as ‘the measurand problem’: whereby two ostensibly identical measurements are found to differ on a more fundamental level. In some applications, this can go further—a specific quantity may be expressed in different ways by different user communities. These differences can impact validation as illustrated below.

For example, Cloud Top Height, Pressure, and Temperature are related ECVs that describe the elevation of a cloud (and will be referred to as CTH, CTP, and CTT, respectively). The conversion between CTT, CTP, and CTH utilizes profiles of atmospheric temperature and geopotential height. Those can be obtained from radiosondes or reanalysis, such as ERA5 (Hersbach et al. 2020), but both use pressure as a vertical coordinate. As pressure decreases exponentially with height, symmetrical distribution of measurement differences in CTT or CTH will be distorted by the conversion. This subtlety is typically ignored, with the largest discrepancy reported alone as if it were a standard uncertainty. This incorrectly represents the tails of the difference distribution.

There are also cases where a single physical concept can be observed in multiple manners, but these are inconsistent on a fundamental level. Active sensors detect CTH as where the density of hydrometeors sharply increases at the edge of a cloud. Passive sensing observes radiance emitted throughout the cloud such that the CTT reported is a weighted average of the temperature profile within the cloud. (The weighting function is sometimes known as the ‘averaging kernel’.) As explained in Povey and Grainger (2015), that CTT is typically warmer than that at the physical top. Hence, validation of passive against active CTH must apply a correction to achieve a ‘like-to-like’ comparison; typically assuming an adiabatic lapse rate (see Sec. 4.6 of McGarragh et al. 2018 or Sec. 2.3.1 of Grosvenor et al. 2018). Thus, the validation of CTH is more of an inter-comparison than a strict validation against a reference observation. Nonetheless, this remains an invaluable method to demonstrate that the passive observation is fit-for-purpose.

SST reference measurements are a good example of where measurand differences must be considered. Satellite SST typically corresponds to the skin temperature (exact depth depends on the spectral range used). In-situ reference SST measurements come from a variety of instrumentation which measure at different depths (O’Carroll et al. 2019). Ship-borne radiometers, for example, will typically measure the skin temperature, whereas buoys within the international Argo programme capture near-surface temperatures (within the top 5 m). Promisingly, there have been concerted efforts by the community to characterize the uncertainties introduced by the different sampling strategies and harmonize datasets (Donlon et al. 2014; Wimmer and Robinson 2016).

### Independence Issues

Independence eliminates the risk of circular reasoning, where the validation process is compromised because the datasets share common inputs, models, or assumptions. Reference measurements are intended to serve as an objective benchmark for satellite data; thus, any overlap in data sources, algorithms, or processing methods between the two can make it harder to identify biases or inflate the apparent agreement. Ultimately, if satellite retrieval algorithms incorporate data derived from the same sources as the reference measurements, the validation results may be affected. In case of large uncertainties on that data source, this may result in skewed validation results and subsequent loss of confidence in the satellite product. The training of a neural network used to classify sea ice types in active satellite microwave imagery is a classic example here.

However, reference datasets may share sources of uncertainty with the observations being evaluated, thus violating the assumption of independence (of uncertainties) required for the validation process. For example, reference datasets of trace gas concentration are usually derived from ground-based remote sensing techniques. Consequently, these datasets rely on retrieval processes similar to those used for satellite-derived products, leading to comparable sources of uncertainty. A notable exception is the in-situ sampling with balloons or aircraft, which is available only for a few species.

In $\hbox {NO}_2$ retrievals specifically, satellite and ground-based datasets tend to rely on the same absorption cross sections (e.g., Vandaele et al. 1998), or they can have similar vertical sensitivity profiles (as in e.g., TROPOMI HCHO vs. FTIR comparisons of Vigouroux et al. 2020). This can be tackled by using a variety of ground-based instruments with different observation geometries (Verhoelst et al. 2021) and/or spectroscopies (e.g., FTIR and UVVis DOAS as in Lambert et al. 2024). If suitable for the study objective(s), prior harmonization of satellite and ground datasets towards the same model profile is a recommended methodology (Rodgers and Connor 2003). However this can insert correlated errors as discussed further in Sect. 4.2.

A final example of independence issues is the CEOS burned area validation protocol (Boschetti et al. 2009), which recommends the use of higher-resolution satellite products to validate coarser-resolution satellite observations (in this case, the reference data are also satellite products). This procedure has been deployed effectively, such as Boschetti et al. (2019) using Landsat imagery to validate MODIS burned area products. However, the two satellite products have similarities in their processing algorithms (e.g., use of spectral indices such as the Burned Area Index) and underlying assumptions, for which the resulting uncertainty cannot be quantified by such assessments.

In all three of these examples, the selected reference datasets are regarded as the most practical options for inter-comparison, either due to the lack of viable alternatives or because alternative approaches are unable to satisfy the requirements of validation. However, such studies effectively assess consistency between datasets rather than quantifying the uncertainty of a single dataset. While consistency assessment is important, this distinction is often not explicitly acknowledged in the reporting.

### Validation Capacity and Completeness

A significant challenge in validation activities lies in ensuring that the reference measurements encompass both the full range of variability in the satellite data under evaluation and the complete range of any parameters that affect the measurand / quantity being measured. This issue is exacerbated by differences in the spatio-temporal sampling of satellite and reference datasets.

From a spatial perspective, satellite observations are typically global, whereas the spatial distribution of reference datasets is often constrained by logistical factors, leading to undersampling in certain geographical regions, biomes, or environmental conditions. Temporal coverage and completeness pose additional difficulties, as reference datasets are often collected seasonally or are limited to specific operational periods. Furthermore, older measurements or historical datasets might be incomplete, sparse, or inconsistent. In some cases, the data may not have been digitized or preserved properly, making it difficult to access or compare with satellite observations, as discussed in Sect. 2.2. Finally, reference networks continuously evolve over time which changes the temporal sampling and which could lead to erroneous trends if used to correct/harmonize data. Several examples are presented here across different ECVs to show the widespread nature of this challenge.

#### ***Aerosol Properties***

**Fig. 1:**
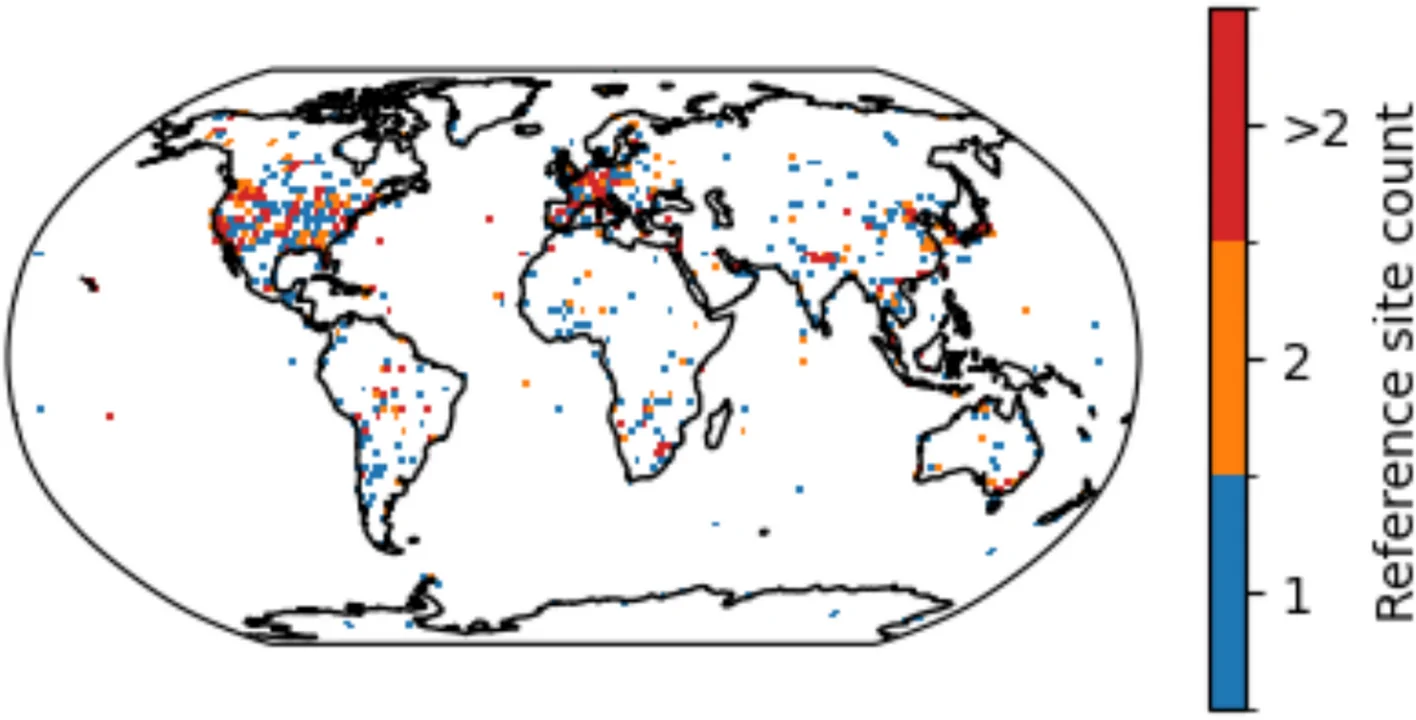
The global density of surface reference sites (on a $2^\circ \times 2^\circ$ grid) from six observational networks: AERONET for aerosol optical depth, RadCalNet for surface reflectance, SURFRAD for surface radiation budget, MPLNet for the vertical distribution of aerosols, FLUXNET for the carbon/water cycles, and TCCON for trace gas concentrations

Surface reference measurements of aerosol properties tend to be concentrated in Europe and the continental USA (e.g., EBAS, https://ebas.nilu.no). Consequently, these observations are most representative of anthropogenic pollution dominated by nitrates, which will differ from pollution dominated by sulphates or even natural aerosols. As another example of reference data, we discuss measurements by the Aerosol Robotic Network (AERONET), an international network of sun-photometers that provide highly accurate observations (via the Direct Sun Algorithm) of the optical depth of aerosols along the line of sight to the sun (Holben et al. 1998). While measurements of AOD from AERONET sites (https://aeronet.gsfc.nasa.gov) have better global coverage than many reference networks, large regions are still undersampled: Northern Africa, Northern Asia, and the oceans. This is illustrated in Fig. 1, alongside a selection of other networks. The location of measurements sites is constrained by resource availability, access, and political considerations. These choices may influence the final ECV product in subtle ways as they affect where scientific development is focused and what it overlooks.

Spatial coverage of the AERONET network has also changed with time, with reduced spatial sampling going back through the data record up to the launch in 1992 (Holben et al. 1998). In particular, and rather obviously, AERONET cannot be used to assess satellite products before that time.

#### ***Land Surface Temperature***

**Fig. 2:**
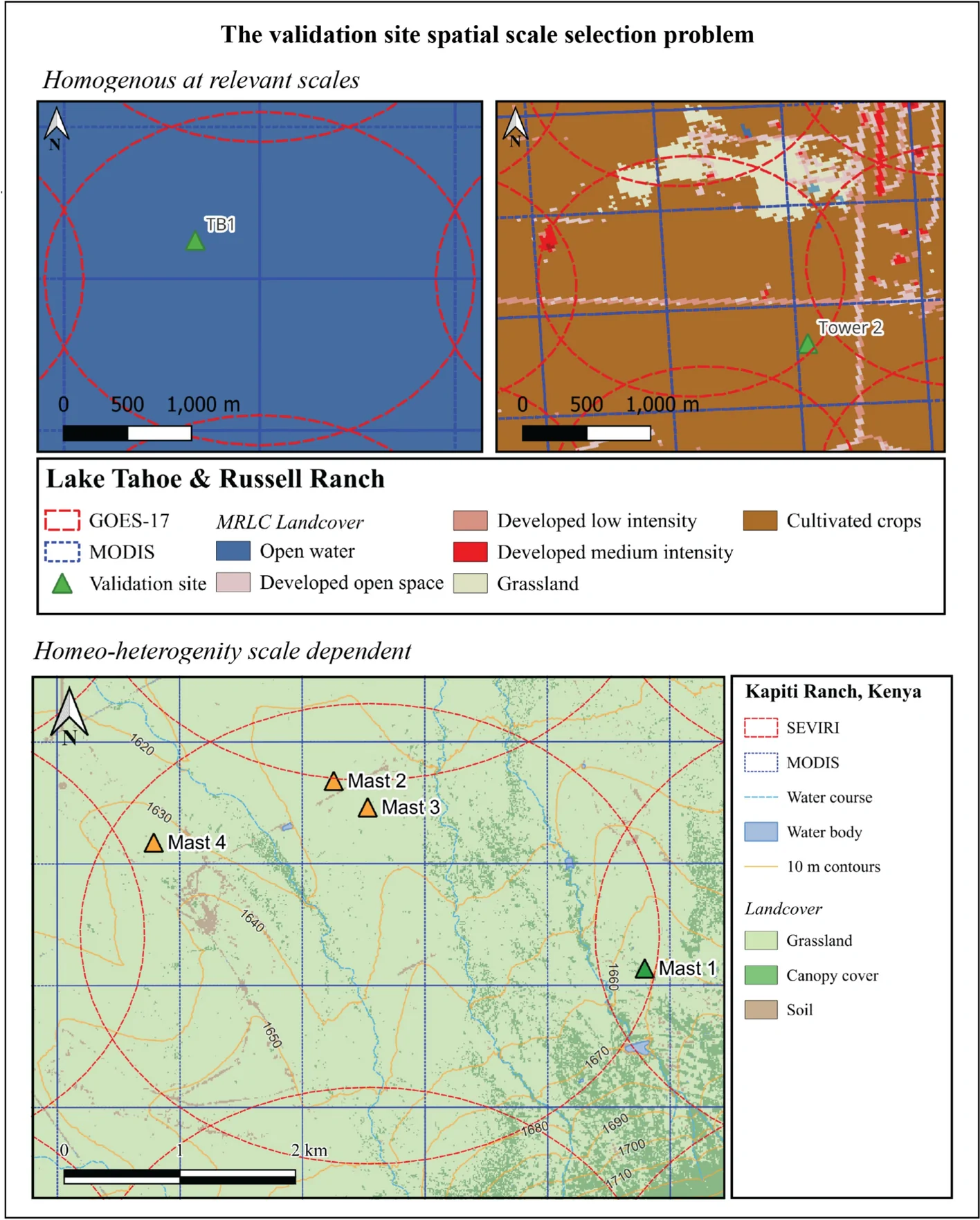
Satellite (MODIS and SEVIRI/GOES) pixel sizes over classical LST calibration sites (top left: Lake Tahoe, USA; top right: Russell Ranch, USA) compared to a calibration site from a region of the world typically not used as such (lower: Kapiti, Kenya). US sites landcover data from the Annual National Land Cover Database (NLCD) Collection 1. Kenya site land cover data from Dowling et al. (2022)

Validation of satellite LST is predominantly conducted over homogeneous environments such as deserts and lakes (Göttsche et al. 2016; Hook et al. 2020) because of the challenges in capturing spatially representative reference LST in heterogeneous environments (Guillevic et al. 2018; Malakar et al. 2018). This is demonstrated in Fig. 2, which shows satellite pixel sizes over long-term LST calibration sites compared with a more heterogeneous site in Kapiti, Kenya. Kapiti is about as homogeneous as a savannah site can be, and yet due to factors such as pixel size and the nature of the landscape (grasslands with scattered and isolated trees and soil), to validate at the pixel scale requires evaluation of all components, which is challenging logistically. If we do not carry out such work then the potential biases of any given product over a landscape with sub-pixel scale heterogeneity remain undetermined. This may lead to an underestimation of overall satellite bias, since validation studies conducted in heterogeneous environments report higher and more variable biases than those observed in homogeneous environments (Duan et al. 2019; Martin et al. 2019; Dowling et al. 2022). This can also result in misunderstanding of LST behaviour in heterogeneous environments. For example, there have been no studies which validate satellite LST products in urban environments without additional assumptions (e.g., downscaling). In practice, this means that we have no understanding of product performance over such environments despite the importance of LST for understanding urban phenomena like the urban heat island effect (Hulley et al. 2019). It is important that validation of a variable covers all types of landscapes, at a spatially relevant scale, where that variable is used for scientific research.

#### ***Sea Ice Thickness and Snow Thickness***

Many field campaigns can only be conducted in particular seasons, especially in the polar regions or where there are strong monsoons. In-situ measurements of sea-ice thickness or snow depth on sea ice are most practical during local spring and summer, but these can be conditions where satellite data are known to be unreliable (in this example, due to melting conditions). Airborne campaigns, such as Operation IceBridge (OIB), typically are conducted in daytime conditions for safety reasons and have therefore almost exclusively occurred during spring as shown in Tables 13 and 14 of (MacGregor et al. 2021). The sampling of validation data must be clearly communicated within data quality reports as it impacts the fidelity with which seasonal and other cycles can be represented. Furthermore, in dynamic systems such as snow/ice evolution, a validation during one part of the cycle does not give credible results for other parts of the cycle (Rostosky et al. 2018; Zhou et al. 2021) and, crucially, will not capture all-year accuracy.

For example, sea ice must meet certain requirements to carry ice mass balance buoys or buoys that measure snow accumulation. The consequence is that almost all buoys are installed on so-called multiyear ice. Observations of sea ice and snow thickness from such buoys are therefore biased towards multiyear ice and do not represent seasonal sea ice (e.g., Zhou et al. 2021; Rostosky et al. 2018). Seasonal sea ice covers an increasing fraction of the Arctic Ocean (Kwok 2018), so that errors in its observation will become more consequential over time.

The limited completeness of reference data also impacts the ability to develop, train, and/or evaluate new algorithms (e.g., Zhou et al. 2018; Braakmann-Folgmann and Donlon 2019). This can be obscured when a product is used to fill gaps in other observations. For example, the derivation of ice surface temperatures in the mixed ice-water zone (where in-situ observations are unavailable due to the dangerous conditions; Zhang et al. 2020) relies on satellite and airborne observations in other conditions to devise a workflow that can be applied to airborne inputs alone. Therefore, the surface temperatures derived are only based on reliable data for the summer months, an issue not taken forward into the analysis.

#### ***Sea Surface Temperature and Salinity***

Historic SST records are limited spatially to the ships’ tracks and navigable waters, with frequent sampling across the established trade routes but sparse sampling elsewhere. In recent years, due to the international Argo project and other buoy initiatives, near-surface sea temperatures and salinity are two of the most routinely sampled variables: currently (2024) the Argo project collects 13,000 data profiles monthly (400+ a day) (Argo Program Office 2024). However, no routine measurement systems exist in seasonally ice-covered waters near the poles, unlike in the open, ice-free ocean where buoys can drift freely without being destroyed or where floats can surface without being damaged by bumping into ice. The spatial coverage of evaluation data for these two ECV quantities is therefore biased to ice-free waters, which has implications for producing diagnostic quality indicators.

Indeed, polar regions’ observations are generally mostly of an expeditionary nature and are much more sparse. Upward looking sonar (ULS) measurements carried out in the Arctic Ocean from submarines as a by-product of security monitoring of that region (Wensnahan and Rothrock 2005) or from fishing or whaling boats reporting on ice, weather and oceanic conditions in polar waters (Worby and Comiso 2004; Divine and Dick 2006) are two exceptions, among others.

#### ***Clear Sky Bias***

A final broader example of validation capacity and completeness is “Clear-sky bias”, namely the phenomenon whereby many observations of the Earth system can only be conducted in the absence of cloud because the cloud obscures the signal of interest (and is related to the issue of day vs. night observation). These data are therefore “clear-sky” observations only, which introduces an unavoidable bias to variables that differ in the presence of clouds. Multiple ECVs are affected by this issue including (i) surface temperatures, which will typically be larger under direct solar illumination than diffuse light (i.e., cloudy sky) (Ermida et al. 2019; Karagali et al. 2022; ii) evaporation from land, which is driven by the land surface temperature (Pan et al. 2024); and (iii) aerosols which may grow in size near clouds due to increased humidity. In some cases, the bias appears only within the evaluation of an ECV quantity, such as the assessment of sea ice concentration using optical or thermal infrared satellite observations (Kern et al. 2022). In cases where the presence of cloud is correlated with a perturbation to the measurand, this issue overlaps with the representativeness issues discussed in Sect. 3.4.

### Representativeness Mismatch

The aim of this subsection is to explore a fundamental difficulty with using reference datasets for validation: the unavoidable spatio-temporal mismatch between satellite and reference data. When measuring a continuous field, any given observation can only sample the population of behaviours that can occur. This applies to both the values that could be reported and the relationships between such values, such as derivatives or correlations. Any bias in the sampling will produce errors.

Taking Low-Earth Orbit satellite observations as an example, the spatial representativeness of a single observation is determined by the sensor’s field-of-view (FoV) and spatial resolution, while its temporal representativeness is limited to the brief integration period as the satellite passes overhead. For an entire dataset, the satellite’s orbit dictates spatio-temporal constraints, such as overpass and revisit times. Similarly, reference measurements used for validation are subject to location-specific and methodological constraints, which limit their spatio-temporal representativeness.

**Fig. 3:**
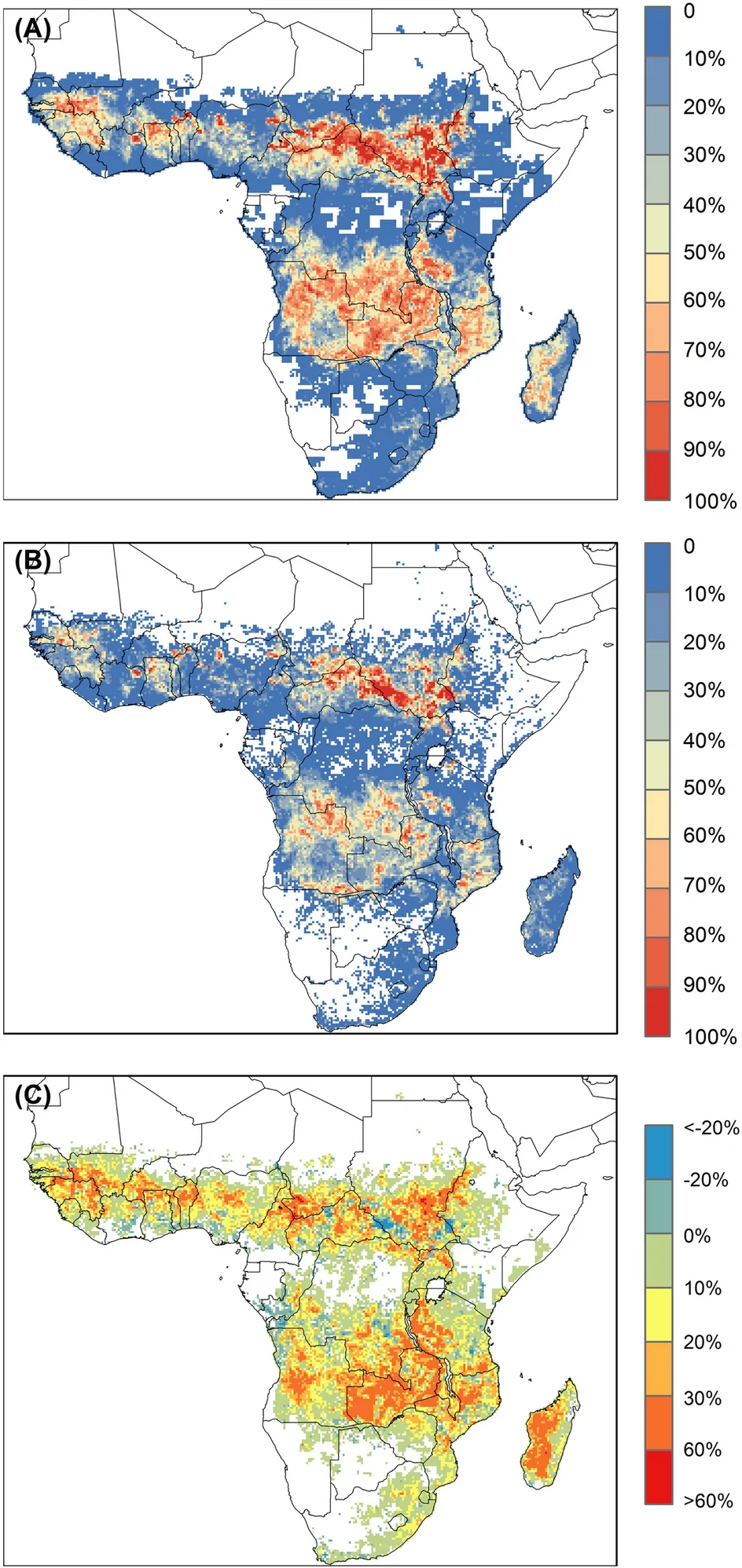
Area burned in 2016, expressed as the fraction of each $0.25^\circ$ grid cell according to (**a**) FireCCISFD11, with a resolution of 20 m; (**b**) GFED4s, with a resolution of $0.25^\circ$; and (**c**) the difference of fractions between them (FireCCISFD11-GFED4s). White indicates a lack of active fires detected by MODIS sensors in 2016. Reproduces Fig. 1 from Ramo et al. (2021)

Differences in spatial resolution can be particularly problematic in the validation of classification data. For example, burned area products identify the area of land surface that has been affected by fire by, say, detecting a darkening of the land. This could cover any scale, from small areas of a few metres to continental. Satellite imagery is comprised of pixels with a set size, dependent on the sensor and pixel location. Burnt patches smaller than that scale are likely to be undetectable and, in practice, detection requires multiple adjacent pixels (further increasing that size). As a result, imagers with different resolutions often report varying burned areas for the same location and time, as illustrated in Fig. 3. While some of this is due to sensor sensitivity, resolution presents a challenge for validation of burned areas. Multiple studies (e.g., Ramo et al. 2021; Andela et al. 2019) show that satellite burnt area products frequently overlook small fires recorded by ground surveys. Indeed, Millington et al. (2022) found that up to 60% of anthropogenic fires were undetected by remotely sensed burned area satellite products, as their size fell below the minimum burned area detectable by satellites. When validating a satellite measurement of discrete events, it is thus important to subdivide the results by the scale of the reference event in order to quantify detection biases in the satellite. This issue is amplified by the limitations of spatial coverage discussed in Sect. 3.3.

**Fig. 4:**
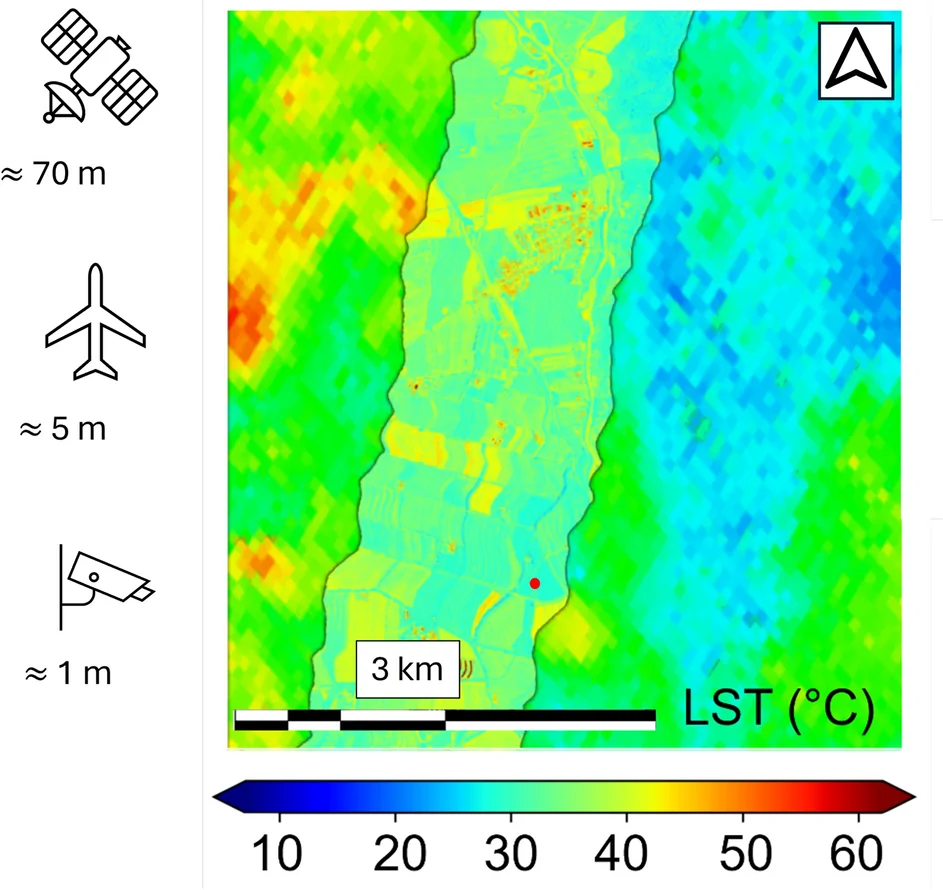
Representation of scaling effects, showing a ground-based observation (red dot) within the airborne swath within a satellite image. The data shown are LSTs captured during the joint ESA-NASA Temperature Sensing Experiment (NETSense) conducted in Grosseto, Italy, in 2019. Airborne observations are taken from NASA’s HyTES sensor, whereas the satellite observations are from the ECOSTRESS satellite. The respective pixel sizes are provided to the left of the image. Image adapted from Wooster et al. (In Review) with permission

Airborne campaigns play a unique role in addressing resolution differences by providing an intermediate spatially resolved observation between point-based reference measurements and satellite observations. This is demonstrated in Fig. 4, which shows ground-based, airborne, and satellite LST imagery.

Field-of-view differences between satellite sensors and reference measurements present additional challenges for validation. For instance, AOD retrievals from AERONET and MODIS (Moderate Resolution Imaging Spectroradiometer) differ significantly in their observational scales. AERONET’s measurements sample a narrow atmospheric path along the line-of-sight to the Sun, with a horizontal extent that varies with solar zenith angle. This horizontal extent can range from nearly zero when the Sun is directly overhead to up to 5.5 km if the Sun is low at the horizon (assuming a zenith angle of $70^o$, and most of the aerosol is in a 2 km thick boundary-layer). In contrast, MODIS Dark Target satellite retrievals (Remer et al. 2005) are based on 20×20 aggregates of pixels that individually have a nominal 500 m field-of-view, but due to off-nadir viewing angles may represent a total area as large as 20×45 $\hbox {km}^2$. Such discrepancies are particularly impactful on validation studies in regions with highly variable aerosol density, such as those near plumes from fires, volcanic eruptions or industrial sources. Different types of aerosol measurements - such as AOD, Ångström exponent, or single scattering albedo - of the same atmospheric volume will be affected differently by these FoV issues (Schutgens et al. 2016a, b).

Angular mismatches, such as differences in the viewing angles of the satellite and reference instruments, can additionally introduce biases due to anisotropic properties of the surface or differing atmospheric path lengths. For ECVs derived from surface reflectance products, extensive research has been conducted into correction of this anisotropy and methods of correcting it (for example, bidirectional reflectance function, BRDF). However, for other ECVs such as LST, this is currently an unresolved problem that can have significant impacts, as discussed in Cao et al. (2019).

Finally, it is important to highlight that mismatch between the spatio-temporal representativeness of two datasets may be a particular problem for historic datasets, particularly when these datasets were not collected with the primary objective of satellite validation. While modern satellites provide continuous, high-resolution measurements, historical observations were frequently catalogued at coarser temporal scales, such as daily or monthly intervals. Historic measurement locations may also lack precise georeferencing or may have changed over time (e.g., station relocation or changes in measurement platforms), which makes it challenging to match ground observations with satellite data spatially. These discrepancies complicate direct comparisons. As discussed in Sect. 2, these historic datasets can be highly valuable in extending the coverage of the validation activity and therefore efforts should be made to address representativeness mismatch issues.

### Potential Solutions

A number of the issues discussed in this section result from poor communication of the variable and its properties. The most important way to minimize the limitations of validation datasets is to clearly communicate in the literature precisely what is measured and the assumptions made in that process. This can be used to minimize measurand and independence issues in particular.

Looking ahead, the availability of reference measurements (validation coverage/capacity) can be greatly expanded by increasing the size of networks and continued commitment to maintain those networks. Network expansion and design should be informed (when not driven) by specific validation needs and identified coverage gaps. Support and funding for airborne campaigns should be maintained for their unique capability to collect data over remote and inaccessible regions and the intermediate resolutions they provide.

Efforts should continue to develop, as far as practical, all reference datasets towards FRM principles, particularly historic measurements. A good example of this is the EU-ESA St3TART project, which aims to prepare a roadmap for operational provision of FRMs for topographic products. Such a roadmap should identify the barriers to applying FRM principles within the datasets as this can drive development of potential FRM-compliant technologies and techniques for validation, as was demonstrated by the sea ice community with the Sea Ice Measurement System (SIMS) (Da Silva et al. 2023).

More broadly, it is important that space agencies and national science centers support dedicated validation campaigns, mirroring the call in Kahn et al. (2023) for systematic sub-orbital observations. These should measure, as closely as possible, the same quantity as that to be validated. The insistence that validation campaigns individually achieve scientific impact, while understandable, increases the uncertainty in the long-term measurements that underpin ECV evaluation by focusing reference observations on ‘interesting’ events. This biases the sample of reference data and tends to overlook routine or inconvenient environments. The recent efforts of ESA with respect to Aeolus and EarthCARE validation and the longer-term combined efforts of ESA and EUMETSAT with respect to Sentinel-3 and Sentinel-6 missions are encouraging. We consider these examples of good practice.

## Challenges and Limitations in the Validation/Comparison Methodology

**Fig. 5:**
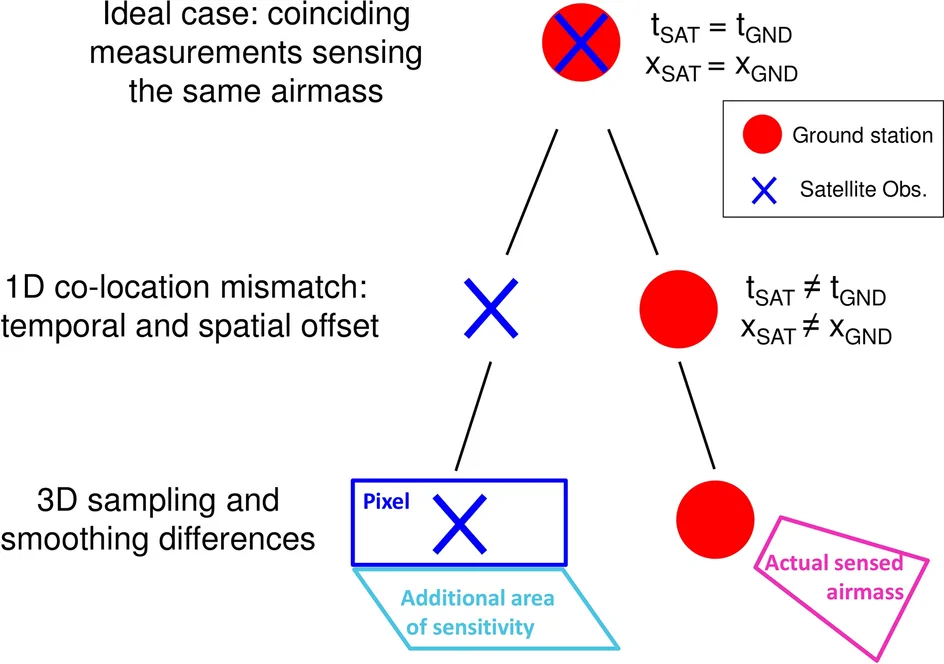
The issue of co-location is not limited to finding satellite and reference measurements with sufficiently similar nominal locations and measurement times, but also includes the impact of differing resolution and perhaps some (even more implicit) sensitivity difference aspects. Image reproduced with permission from Verhoelst et al. (2015)

Virtually all satellite validation work relies on the creation of harmonized and co-located pairs (or triplets/multiplets) of satellite and reference measurements, from which quality indicators (bias, dispersion, etc.) are derived. As touched upon in Sect. 3, the validation and comparison methodology must therefore—to the extent possible—address the differences between the satellite and reference observing systems, in particular regarding spatio-temporal representativeness (Sect. 3.4). This challenge is conceptualized (for the case of atmospheric composition data) in Fig. 5, which details that instead of comparing two point-like measurements that coincide in both space and time, reality is much more complex: not only do the nominal measurement locations and times differ, also the actual area/time of sensitivity will be different, and more often than not, neither the satellite pixel coordinates nor the instrument location capture the full sensitivity of the measurements. If the ECV under study is variable at these scales, this inevitably introduces mismatch-related error.

In practice, the harmonization and co-location criteria are designed to minimize mismatch errors while still yielding sufficiently large sample sizes to construct robust statistics (e.g., for bias estimation). While the latter constraint usually excludes the possibility of a “perfect" co-location, most ECVs describe the evolution of a dynamical system and exhibit substantial spatio-temporal correlations, such that much can be learned from a comparison of two datasets that refer to slightly different times and locations. Still, it is important to be aware of the complete uncertainty budget of a comparison, including measurement uncertainties on satellite and reference data, additional uncertainties introduced by harmonization steps, and uncertainties due to remaining, irreducible, co-location mismatch. When these additional uncertainties are of greater magnitude than those of the dataset to be validated, the value of the validation becomes questionable.

Below, we discuss in more detail the complete uncertainty budget, the need for harmonization, the (irreducible) co-location mismatch, and approaches to optimize the comparison.

### Constructing a Complete Comparison Uncertainty Budget

Constructing a complete comparison uncertainty budget for satellite validation involves systematically quantifying all sources of uncertainty that may affect the comparison between satellite observations and reference measurements.

In theory, this process could be completed using a three-step approach:

1. Identifying and quantifying uncertainties related to the reference measurements themselves (discussed in Sect. 2). These include stochastic measurement noise, calibration effects, and the influence of auxiliary inputs or algorithm assumptions.
2. Identifying and quantifying uncertainties from spatial, temporal, and angular mismatches and approaches to address these (for example, using modelling or higher resolution imagery).
3. Propagation of uncertainties through the comparison process, often using Monte Carlo simulations, analytical uncertainty propagation, or covariance analysis techniques to capture their combined effects.

The resulting uncertainty components would then be aggregated into a total uncertainty budget, typically expressed as a standard deviation or confidence interval.

In practice, this process is significantly more challenging due to the issues raised throughout this paper. For example, there may be incomplete characterization and knowledge of uncertainties as discussed in Sect. 2.3, which will generally result in omission of uncertainty contributors and result in underestimation of the total uncertainty. There may be independence issues not accounted for, as discussed in Sect. 3.2, which result in correlations between uncertainty sources, such as shared biases in atmospheric corrections or geolocation inaccuracies. Assumptions made in the representativeness of the reference datasets may be incorrect that result in unquantifiable uncertainties overlooked in the budget. An example of this could be drift in a historic station location over time that was not recorded in metadata, thus not captured in the uncertainty budget. Estimating co-location mismatch uncertainties is highly difficult as it requires a detailed understanding of both the measurement systems and the spatio-temporal variability of the ECV under study (Sect. 3.4). Finally, there may be more conceptual challenges, such as differences in the measurand, that are harder to capture the uncertainties of. While this list of challenges is daunting, awareness of potential issues is the first step, and incremental progress towards a complete uncertainty budget is being made for many ECVs. Eventually, the aim of the validation should be to verify whether the uncertainty budget is closed, i.e., that the observed distribution of differences is consistent with the total estimated comparison uncertainty budget. Pioneering work in this direction has been published by Verhoelst et al. (2015) and Sayer et al. (2020), and is the topic of Verhoelst et al. (2025) in this special issue.

### Harmonization between Satellite and Reference Datasets

Harmonization is essential when comparing two datasets as they will almost always have different spatio-temporal representativeness, on both the local (single measurement) and global (full dataset) scale. While such harmonization can eliminate/reduce significantly the mismatch errors, it is essential to be aware of the implications and additional uncertainties introduced, as detailed below.

First, the essential step of creating a common subset (through spatio-temporal co-location) of both datasets (and constraining the comparison to those data) limits the evaluation of satellite-observed ECVs to a spatio-temporal sampling that is usually far more sparse than the original product. This subset may not be representative of the original dataset, depending on the validation capacity of the reference dataset, as already discussed in Sect. 3.3.

Second, there is the issue of how to compare individual observations that may have very different spatio-temporal resolutions. Upscaling and downscaling approaches are common, with different approaches advised depending on the ECV and location. Criteria for a satisfactory co-location will vary from ECV to ECV, depending on spatio-temporal correlations that exist in the actual ECV fields. Surprisingly little research has been done on these spatio-temporal correlations (Anderson et al. 2003 and Sparling et al. 2006 are some examples). Co-location criteria themselves (i.e., maximum separation in space and time of satellite and reference data) are often determined through trial-and-error to achieve the best agreement between satellite data and reference data. In any case, a representativeness error will be introduced by the harmonization, e.g., through the loss of information on individual satellite pixels or through the use of auxiliary data with their own uncertainties. These errors will exhibit both random and systematic behaviour, demanding a careful approach to quantifying their statistics to account for their spatio-temporal correlations.

Differences in temporal sampling or resolution between satellite and reference instruments are a particular concern for ECVs with a strong diurnal evolution. For example, the observation of tropospheric and stratospheric $\hbox {NO}_x$ is challenging as the gases have short photochemical lifetimes (of the order of hours) and—for the tropospheric component—a strong diurnal evolution in their (anthropogenic) emissions (e.g., rush hour traffic). When high $\hbox {NO}_x$ scenes are masked by clouds (Compernolle et al. 2020), the validation approach must ensure either very tight temporal co-location or use conversion factors to harmonize the temporal coverage. The latter approach is adopted when comparing satellite stratospheric $\hbox {NO}_2$ observations, obtained at low-to-moderate solar zenith angles, to ground-based reference measurements, obtained during sunrise and sunset with twilight zenith-scattered-light instruments (Verhoelst et al. 2021). These $\hbox {NO}_2$ column measurement conversion factors are based on a photochemical model representing the full diurnal cycle as a function of latitude and time-of-year, but introduce additional uncertainty to the validation.

**Fig. 6:**
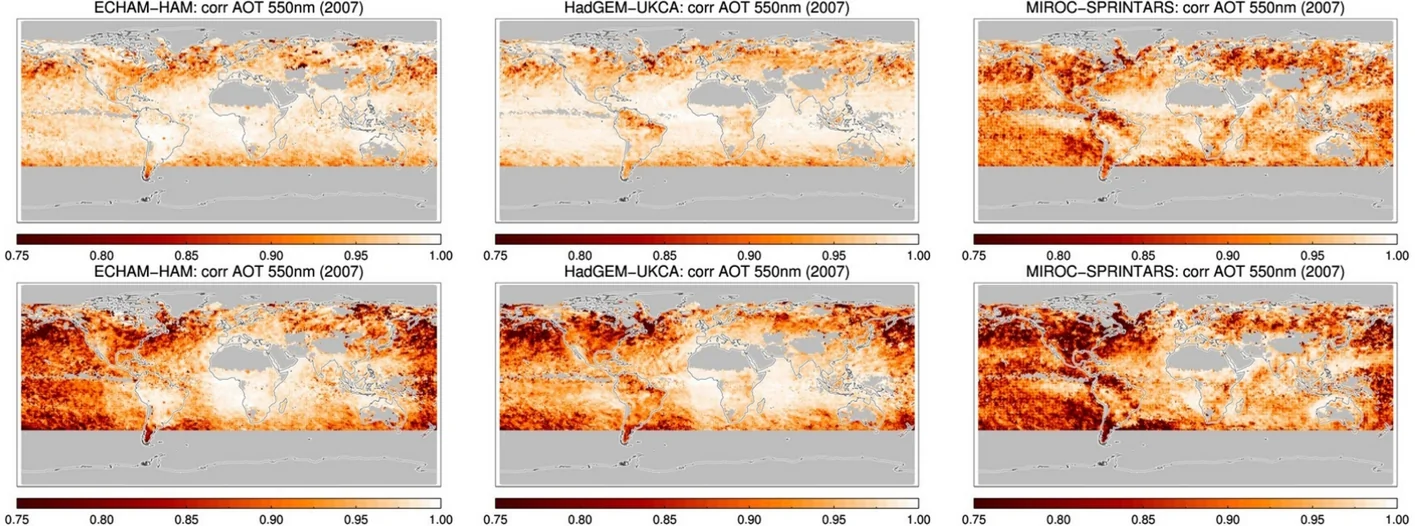
Correlation between model AOT at the time of MODIS-NRL Aqua observation and a 24 h model AOT average. Top: 24 h model average uses overpass time as the center for the 24 h average; bottom: 24 h average defined according to UTC. Image reproduced with permission from Schutgens et al. (2016b)

A more problematic case of harmonization of temporal differences arises when evaluating model data with satellite data. When daily model data (centered on UTC noon) is compared with satellite data from single overpasses on that day, the model will appear to perform worse near the dateline than near the central meridian (Schutgens et al. 2016a). This effect disappears if the model is instead averaged around a small time-window centered on the overpass time, as shown in Fig. 6.

Third, the harmonization step typically includes operations to align measurands, units, and underlying assumptions on the state of the ECV. When these require the use of auxiliary data, additional uncertainties seep into the comparison, and independence between satellite and reference measurements may be lost (as already touched upon in Sect. 3.2).

Finally, historical datasets often require processes like homogenization (adjusting for inhomogeneities over time) or reconstruction (e.g., using proxy data). The techniques used for these processes can introduce uncertainties, making it harder to compare historical data directly with satellite measurements.

### Irreducible Co-location Mismatch

Even after harmonization and an application of “optimal" co-location criteria, discussed in depth in Loew et al. (2017), some mismatch errors will remain, in particular if (1) these criteria are relatively relaxed so as to ensure a sufficiently large number of pairs to obtain robust statistics, (2) the spatio-temporal distribution of the ECV has strong variability at the scale of the co-location (which may be a typical property of the ECV or the result of some localized event), and (3) the measurement systems have complicated spatio-temporal sensitivities to the measured ECV, not well represented by the nominal pixel area or site location. Quantifying these irreducible errors is challenging, in particular because often only limited knowledge exists on the fine-scale variability of the ECV and on the more subtle sensitivity properties of the measurements. This highlights the importance of validating uncertainty information on mature ECVs, as one of the few means of quantifying the behaviour of errors of this nature.

### Potential Solutions

Considering the harmonization and co-location process, thoroughly and precisely describing the aggregation, filtering, and interpolation methods used in the generation of data is essential to determining the nature of errors expected when comparing two measurements. Filtering criteria, such as thresholds, and the rationale for their selection are too often omitted from publications, even in technical reports.

Ideally, the co-location criteria should strike the optimal balance between small mismatch errors and a sufficient number of comparison pairs to allow an accurate determination of the quality indicators, but as criteria are often derived by trial-and-error, dedicated studies aimed at quantifying this optimum are needed. An adequate design of field experiments and airborne campaigns that are specifically dedicated to evaluate satellite measurements is mandatory in this regard as well; see also Kahn et al. (2023).

Errors introduced by representativeness issues that remain after (or are introduced by) the harmonization process need to be understood and preferably become part of the comparison uncertainty budget. Methods exist to estimate these mismatch uncertainties but their use is not yet commonplace. These include the triple co-location analysis (e.g., Virtanen et al. 2018; Gruber et al. 2016), and the use of models and/or other high-resolution data (e.g., from field or airborne campaigns) of the ECV to quantify the mismatch errors at the scale of the co-location (e.g., Verhoelst et al. 2015; Schutgens 2020). Ideally, this includes a metrological analysis of the true spatio-temporal sensitivity of both measurement systems to fully account for the representation differences (e.g., Lambert et al. 2012).

To help with construction of a complete comparison uncertainty budget, we again advocate metrological methods such as the uncertainty tree to identify potential contributors. In some areas of study, methods have been developed to address some of the issues identified in Sect. 4.1. In the case of measurand differences, one of the datasets may be able to be mapped onto the other or manipulated to more closely resemble the other. However, this process will introduce additional uncertainties that have to be considered. To estimate representativeness uncertainties, scientists may benefit from the use of external datasets (e.g., high-res model or campaign data).

Finally, a good option to acknowledge potential blind spots or mischaracterizations in the uncertainty estimation is the "plus zero uncertainty" term (Mittaz et al. 2019; Bulgin et al. 2022). This is an unquantified or unmodelled uncertainty component that is assumed to not contribute to the measurement equation but does contribute to the uncertainty budget. This can be particularly valuable in acknowledging unidentified uncertainty contributors. A means of avoiding human bias within the validation process is to ensure that, at least occasionally, the validation of an ECV dataset is performed by a researcher uninvolved in its creation, as this person will not have a vested interest in the final result and will likely optimize for different practical concerns.

## Recommendations to the Community

Common themes identified as potential solutions throughout this paper are discussed further below.

### Identification of Limitations

As a first step, for any data collector, it is essential for continued improvement of climate observations that efforts are undertaken for systematic identification of limitations within different datasets. This includes all limitations discussed in this paper, from traceability to harmonization of the satellite product and the reference dataset. Without this initial step, there can be no possibility for improvement.

Drawing from the metrological community, an exemplary means of evaluating limitations would be the production of an uncertainty tree (Merchant et al. 2017). This can enable both assessment of the resulting uncertainties as well as identification of inputs which are lacking uncertainty quantification.

The maturity matrix is another good example of a method that can be used to identify limitations in the validation process. This makes use of a systematic approach to identify the maturity of an aspect of the data. In the Copernicus Cal/Val Solution (CCVS) project for example, a pioneering maturity matrix has been developed for use in validation activities. This maturity matrix has been applied to atmospheric composition products of Copernicus Sentinels (Alhammoud et al. 2023, page 51). We recommend further development of the validation maturity framework as pioneered in CCVS. The maturity assessment frameworks of EDAP (EDAP 2022) and CEOS-FRM (Goryl et al. 2023) can also serve as inspiration.

One can make a further distinction between (a) the maturity of the validation process itself according to state-of-the-art protocols or community practices, and (b) the completeness of the validation as performed for a given product. A first step towards (a) is for community best practices to be identified, further discussed in Sect. 5.5.

However, we must emphasize that the maturity rating communicated to the user does not comment upon the merit of the data. If the only data available have no uncertainty and no ability to estimate it, that does not mean the data lack value, it just comes with caveats for the use and application of that data. The maximum maturity level may be impossible or impractical to achieve for certain ECVs (as currently defined and measured).

### Communication of Limitations in the Validation Process

As discussed throughout this paper, identification of the limitations in the validation process must be accompanied by effective communication of these limitations. Users will vary in their understanding of what validation data means for their desired use, and in their technical ability to handle the validation data. In particular, a focus must be placed on conveying the scope and applicability of the validation work carried out. As discussed in Sect. 3.3, practical and logistical concerns may limit the collection of validation data to specific conditions, such as a single season or surface type. It is important to convey the domain/extent of the validation data to users so they can make informed decisions about how to apply those results to other circumstances. The communication of limitations is not intended to restrict or diminish a dataset but rather enable the widest appropriate use, and facilitate discussion about what more work needs to be done.

It is therefore important to have a means of communicating to a user that, while the stated uncertainty for the product may be, for example, 10%, the temporal and spatial resolution of that uncertainty is not necessarily uniform and there may be error correlations. A good framework within which to evaluate and state the domain and conditions over which validation results apply, to inform about whether error correlation scales are included, and to highlight other issues as described, is once again the maturity matrix.

However, we again emphasize that the maturity matrix cannot be considered a measure of merit. For example, as discussed for sea ice, there can be barriers to extending the validation data beyond a specific set of conditions. In such cases, the maturity of the assessment may never advance beyond a certain level. To avoid mis-interpretation that could have impacts downstream (e.g., on the availability of funding), care must be made to accompany the maturity matrix with suitable documentation explaining the reason for this.

### Data Storage and Public Repositories

Robust data storage is essential to ensure the preservation and discoverability of validation data. This is essential both for the principle of reproducible science and to enable reprocessing in response to future discoveries. While the discoverability and dissemination of satellite data have greatly advanced in the last twenty years, the same cannot be said for the validation of the datasets that underpin this primary data. Where they do exist, many cal/val datasets are often left in the limbo of grey literature, stored on inaccessible servers or buried in dense technical reports in non-machine readable formats.

Example solutions for the issues raised above have been identified in the atmospheric science community, who have developed resources such as the ‘Homeless’ data portal (ATMO-ACCESS 2023) and contributed to ESA’s Earth Observation Validation Data Centre (EVDC) (https://evdc.esa.int/). However, as campaigns often collect data across multiple ECV domains, there is a further need for a single platform for multiple ECV communities. We therefore encourage the development of a dedicated cal/val data portal, coordinated centrally by the space agencies, with unified metadata standards, linking of data to its documentation at the source, and guaranteed long-term archiving and cross-referencing support. A potential option for this is the CEOS WGCV Cal/Val portal hosted by ESA (https://calvalportal.ceos.org/). While still in the early stages, and with limitations in both its presentation and use, this portal is recommended for further development as this may be a suitable hub for the storage and dissemination of multiple ECV validation data. In particular, the clear standards and assistive onboarding process would provide a significant step forward in solving the issues raised above. We highlight the need for maturity assessment frameworks and tools, such as the maturity matrix, to accompany these datasets within the portals.

While the CEOS WGCV effort includes a broad collection of cal/val data in different thematic domains, further good examples of dissemination for a specific task is that of the QA4SM effort (https://qa4sm.eu/ui/home) or AERONET (https://aeronet.gsfc.nasa.gov/). Like EVDC, both of these portals enable a user to easily locate and use machine-readable data that are directly relevant to their task. They also streamline the location of key documents and training in best data practices.

As a final consideration around data storage, we highlight the need for scientific communities to make use of these portals to increase validation capacity and coverage. We encourage any field-going party to publish their measurements together with the logical minimum set of metadata, regardless of whether these are FRM-compliant or not. It is essential to include both raw and processed data to facilitate reprocessing in case of future discoveries and potential ongoing uncertainty analysis, for example, as was observed for calibration coefficients in allometric equations used to derive Above Ground Biomass reference datasets (Calders et al. 2022).

### Extension of Station Networks

To address representation issues, we need to extend and build upon existing station networks and establish long-term monitoring stations in under-represented areas, both geographically as well as in terms of research focus. The community therefore needs to make a continued push to secure dedicated, long-term funding for fixed stations globally with multi-national infrastructure that can be built in the global south and other areas of limited observation density. To ensure that stations strive to FRM quality, the support of these stations should include maintenance, regular re-calibration, equipment purchase, and support for local expertise. The ongoing ESA Cal/Val Park Project is an example of best practice of site development, as this site aims to be one which will adhere to FRM principles enabling cal/val opportunities for satellite missions including those from New Space.

A critical consideration in network expansion is determining optimal locations for new stations. Heuristic approaches can be useful, such as ensuring the network captures the full range of natural variability of an ECV and the parameters influencing its retrieval. For example, an aerosol network should include observations representing the natural variability of AOD (‘amount’) as well as across the natural range of particle sizes and compositions, since these also affect satellite retrievals of the AOD ECV. Beyond heuristics, it is recommended that other objective standards for locating sites are developed. Schutgens et al. (2020) proposed using diversity among satellite-derived AOD products as a criterion for developing siting rules, with new sites preferably in areas where satellite products differ greatly.

In addition to fixed long-term networks, dedicated airborne campaigns have a significant role in bridging resolution issues and providing data in remote or otherwise inaccessible regions. As discussed in Sect. 3.3 and MacGregor et al. (2021), these have their own challenges and likely will only capture a snapshot of conditions, but are often the best—and sometimes only—option for satellite-equivalent observations in challenging locations. Continued efforts in this area are critical, and individual communities should highlight their needs.

Extension of observations to address representativeness issues requires exploiting every technology available. Firstly, in situations where cost constraints prohibit network establishment or expansion, well-calibrated low-cost environmental sensors present an alternative option. Chan et al. (2020) demonstrated that, across a range of environmental measures, low-cost instruments can achieve research-grade standards if deployed and maintained carefully. These sensors significantly reduce hardware costs, with much of the build and maintenance able to be performed locally. To strive to FRM standards, these sensors may require more frequent maintenance checks than more robust instrumentation and concerted efforts must be made to ensure regular re-calibration against traceable standards using community best practice. Rigorous reporting practices as well as the retention of raw data are essential to maximize the scientific utility of these datasets.

Secondly, emerging technologies and validation methods, such as drones providing access to remote areas, also offer new opportunities to extend measurement networks. These technologies and associated processing techniques are still maturing and often are not yet FRM-compliant. It is therefore important that the scientific community apply rigorous quality assessment to ensure their reliability. A good example is the SRIX4Veg (Surface Reflectance Inter-comparison eXercise for Vegetation), endorsed by CEOS and conducted as part of the ESA FRM4Veg project. This initiative demonstrated the potential of UAV-based surface reflectance measurements by investigating instrument and operator biases from different groups as well as their respective measurement collection protocols.

### Community Work to Establish Best Practices

As recognized throughout this paper and more generally throughout this special issue, there have been significant and appreciated efforts from scientific communities and space agencies to establish best practices and standardized validation protocols in the atmospheric, ocean, and terrestrial domains. These efforts include the CEOS WGCV community guidelines and protocols, QA4EO, the CEOS interoperability framework, and the FRM4XXX projects (Goryl et al. 2023; Niro et al. 2021; Loew et al. 2017; Banks et al. 2020; Brown et al. 2021; Sha et al. 2024). Relying usually on community practices published in the peer-reviewed literature, these best practice protocols have been well received by the wider scientific community, with significant uptake of the recommended methods and approaches across different ECVs, including LST (Dowling et al. 2022), ozone (Verhoelst et al. 2015), nitrogen dioxide (Verhoelst et al. 2021), ocean colour (Brewin et al. 2023; Ruddick et al. 2024), as well as the development of tools to support user uptake of best practices (NPL 2024). At present however, there are still ECVs that lack such efforts, particularly for areas that are acutely affected by the error effects reviewed in this paper. Instead efforts have, understandably, focused on ECVs where FRM activities are either well established or relatively easy to setup.

For existing activities, we recommend continued efforts to maintain, update, and publicize best practice protocols, accounting for new technologies and validation techniques as they become available. To support this, we also advocate that efforts include training activities to promote this knowledge, such as the recent FRM4SOC (Fiducial Reference Measurements for Satellite Ocean Colour) Training for in situ Ocean Colour Above-Water Radiometry towards Satellite Validation, organized as part of the FRM4SOC-2 project (EU Copernicus FRM4SOC-2 Project / EUMETSAT 2025).

To support maturation of uncertainty information in the more difficult or newer areas of observation, space agencies should support activities where data collectors, data users, and space agencies can share experiences and develop practical steps towards achieving universal best practices. This will require coordination and funding commitments from space agencies and other organizations, as was done with the International Space Science Institute (ISSI) workshop (which led to this special issue). These efforts are essential to maintain user confidence in satellite-derived products and ensure reliable, comparable, and accurate data are available to study our changing climate.

We recommend that, if not done already, producers of best-practice protocols account for uncertainty associated with parameters which feed into derivation of the ECVs alongside the ECVs themselves. As the work in Sect. 2 highlighted, these datasets may have had a less robust assessment of their uncertainty. Where possible, there should be concerted efforts across validation communities to develop best-practice methods to assess quality and judge whether measurements are trustworthy and fit-for-purpose. While we leave exact definitions of these best-practice methods to the specific ECV user communities, when extending to non-ECVs, we suggest starting with parameters that feed into multiple ECVs—or which certain ECVs are particularly sensitive to.

Finally, as discussed in this paper, there will be times where establishment of a best-practice protocol is not yet possible. In these cases, discussion of the different methods and the known limitations and challenges is a vital first step.

## Conclusions

This paper discussed the different challenges and limitations of validating satellite-derived datasets using independent measurements for different ECVs and ECV quantities within terrestrial, ocean, and atmospheric domains. From this discussion, potential solutions and recommendations were presented for users and producers of such products in order to account for these challenges and limitations. While each ECV presents unique challenges, common themes are observed across the different ECVs.

Some issues identified are considered to be pragmatic, such as funding and logistics for the reference measurements limiting spatio-temporal representativeness and completeness of the validation. These may be unavoidable as yet, for example, spatial representativeness being limited by certain sites that are too difficult or dangerous to access using existing technology and techniques. However, the work presented in this paper identified a need for a concerted global push to extend existing monitoring networks where possible, particularly in undersampled areas and measurement regimes. This should be accompanied by exploration of new technologies and techniques to access previously inaccessible regions. Extension of station networks and uptake of new technologies and techniques will require multi-institution coordination and financial support. In the case of the ECVs, we reiterate the importance of establishing reference measurements that capture as much of the range of variability of the ECV, but also of its influencing quantities, as possible. Differences in the spatio-temporal sampling of datasets, such as omissions in locations and seasons, can produce apparent biases as the two methods are observing different populations of the measurand, of its uncertainty sources, or different realizations of the environment. As previously rare climate events occur with increasing frequency, reference observations that only capture the easiest or most common conditions may contain “unknown unknowns”. All of these effects can have consequences downstream in derived values and decision making, including climate model forecasts or predictions, which may have significant financial impacts.

In the specific case of spatio-temporal representativeness, we cannot recommend a specific resolution or sampling for validation activities as relevant resolutions/sampling are different for different applications, such that a single dataset could be almost perfect for one application while difficult to use for another. Assessment must be made by each ECV user community regarding the omissions and urgent actions identified and prioritized. Clear statement of the problem to be evaluated, and the inputs to be used, are essential in making that assessment.

Another pragmatic issue identified in this paper is the need for robust data storage accompanied by accurate and sufficient metadata, alongside data quality control. Examples of successful portals that should be considered good examples to the community have been highlighted in Sect. 5.3, with the CEOS WGCV Cal/Val portal and EVDC considered to be excellent potential solutions provided there is continued community buy-in and multi-institution operational support.

Other common challenges identified are more conceptual within the validation process. For example, as explored in Sect. 3, simply because two datasets contain a variable with the same name does not mean those quantities are directly comparable. There can be differences in the measurand (i.e., the precise nature of what the measurement is sensitive to) that result in “apples-to-oranges" comparisons. Similarly, reference datasets are not always applicable as the controlled conditions of a laboratory may not represent a complex, multifaceted reality.

These issues are compounded when using datasets from archives or data repositories, as the precise nature of the observation process may not be clearly communicated or understood. While metrological techniques, such as the uncertainty tree, can help with this issue, “unknown unknowns” or a lack of confidence with mathematics can generate a substantial barrier to utilizing such methods. The environmental science community should develop a standardized qualitative method of communicating the nature of error sources in a dataset which is appropriate to the level of understanding and the audience. Maturity matrices, described throughout this paper and in particular in Sects. 1 and 5, can provide a formal framework within which to monitor the maturation/development of any dataset without judging the merit of the data. In our view, the ease with which such a matrix can be skilfully interpreted by an inexperienced user makes this a suitable solution. Data generators should produce short guides for inexperienced users highlighting common misconceptions and potential pitfalls, while also making any available training materials easily discoverable at the point of data access. Such matrices should accompany datasets within the aforementioned data portals for ease of use.

Finally, and more fundamentally, while providing (ideally) metrologically traced uncertainties on validation data should be a key aim for scientists, we must acknowledge that some environmental data will always be highly uncertain and some errors will always be unquantifiable. This is the cost of leaving the laboratory and studying the real world. There will always be limitations to the accuracy, utility, and validity of a validation exercise. These will derive from the data to be assessed, the references against which it is judged, and the comparison method(s) used. Some of these challenges can be addressed through better communication between users and producers, the targeting of reference observations at providing a comprehensive view of the domain of the measurand, and (that eternal limitation) more funding. Others are unavoidable and intrinsic facets of the data, which should be embraced, not hidden, in order to make the most appropriate and comprehensive use of the information conveyed within an ECV or ECV quantity dataset. Even if the quality of the data themselves do not change, simply by understanding what was collected, how it was collected, and resulting sources of error would represent a significant advance that improves our understanding of our Earth system.

## Abbreviations

ACRE: Atmospheric Circulation Reconstructions over the Earth
AERONET: Aerosol Robotic Network
AI: Artificial Intelligence
AOD: Aerosol Optical Depth
CCI: Climate Change Initiative
CCVS: Copernicus Cal/Val Solution
CEOS: Committee on Earth Observation Satellites
CEOS-FRM: CEOS Fiducial Reference Measurement Maturity Assessment Framework
CDR: Climate Data Record
CMU: Collocation mismatch uncertainty
CTH: Cloud Top Height
CTP: Cloud Top Pressure
CTT: Cloud Top Temperature
ECV: Essential climate variable
EDAP: Earthnet Data Assessment Project
EO: Earth Observation
ESDR: Earth System Data Record
EUMETSAT: European Organisation for the Exploitation of Meteorological Satellites
FAIR: Findability, Accessibility, Interoperability and Reusability
FRM: Fiducial Reference Measurement
GAIA-CLIM: Gap Analysis for Integrated Atmospheric ECV CLImate Monitoring
GCOS: Global Climate Observing System
ISSI: International Space Science Institute
LST: Land Surface Temperature
MEDARE: MEditerranean climate DAta REscue
NDACC: Network for the Detection of Atmospheric Composition Change
OIB: Operation Ice-Bridge
QA4EO: Quality Assurance Framework for Earth Observation
SI: International System of Units
SST: Sea Surface Temperature
TRL: Technology or Technical Readiness Level
TROPOMI: Tropospheric Monitoring Instrument
ULS: Upward Looking Sonar
WGCV: CEOS Working Group on Calibration and Validation

## References

[CR1] Alhammoud B, Clerc S, Verhoelst T et al (2023) Recommendations for R &D on CAL/VAL methods. deliverable d3.2. Tech. rep., CCVS

[CR2] Allan R, Endfield G, Damodaran V et al (2016) Toward integrated historical climate research: the example of atmospheric circulation reconstructions over the earth. WIREs Clim Change 7(164):174. 10.1002/wcc.379

[CR3] Andela N, Morton DC, Giglio L et al (2019) The global fire atlas of individual fire size, duration, speed and direction. Earth Syst Sci Data 11(2):529–552. 10.5194/essd-11-529-2019

[CR4] Anderson TE, Charlson RJ, Winker DM et al (2003) Mesoscale variations of tropospheric aerosols. J Atmos Sci 60:119–136

[CR5] Argo Program Office (2024) Argo international program. URL https://argo.ucsd.edu/

[CR6] ATMO-ACCESS (2023) Homeless data portal. URL https://www.atmo-access.eu/the-atmo-access-virtual-access-portal-has-been-launched/, accessed on Nov 13, 2024

[CR7] Baldridge A, Hook S, Grove C et al (2009) The aster spectral library version 2.0. Remote Sens Environ 113(4):711–715. 10.1016/j.rse.2008.11.007

[CR8] Banks AC, Vendt R, Alikas K et al (2020) Fiducial reference measurements for satellite ocean colour (frm4soc). Remote Sens. 10.3390/rs12081322

[CR9] Bates JJ, Privette JL (2012) A maturity matrix for assessing the completeness of climate data records. EOS Trans Am Geophys Union 93(44):437–452. 10.5194/acp-20-8017-2020

[CR10] BIPM, IEC, IFCC et al (2012) International vocabulary of metrology — basic and general concepts and associated terms (VIM). Joint committee for guides in metrology, JCGM 200:2012. (3rd edition), 10.59161/JCGM200-2012

[CR11] Boschetti L, Roy D, Justice C (2009) International global burned area satellite product validation protocol. part i–production and standardization of validation reference data. Committee on Earth Observation Satellites: Maryland, MD, USA pp 1–11

[CR12] Boschetti L, Roy DP, Giglio L et al (2019) Global validation of the collection 6 modis burned area product. Remote Sens Environ 235:111490. 10.1016/j.rse.2019.111490 PMC7241595 32440029

[CR13] Braakmann-Folgmann A, Donlon C (2019) Estimating snow depth on arctic sea ice using satellite microwave radiometry and a neural network. Cryosphere 13(9):2412–2438. 10.5194/tc-13-2421-2019

[CR14] Breunung T, Balachandran B (2024) Prediction of freak waves from buoy measurements. Sci Rep 14(1):16048. 10.1038/s41598-024-66315-3 PMC11258124 39026003

[CR15] Brewin RJ, Sathyendranath S, Kulk G et al (2023) Ocean carbon from space: current status and priorities for the next decade. Earth Sci Rev 240:104386. 10.1016/j.earscirev.2023.104386

[CR16] Brown LA, Camacho F, García-Santos V et al (2021) Fiducial reference measurements for vegetation bio-geophysical variables: an end-to-end uncertainty evaluation framework. Remote Sens. 10.3390/rs13163194

[CR17] Bulgin CE, Thomas CM, Waller JA et al (2022) Representation uncertainty in the earth sciences. AGU Earth Space Sci. 10.1029/2021EA002129

[CR18] Burgdorf AM, Brönnimann S, Adamson G et al (2023) Docu-clim: a global documentary climate dataset for climate reconstructions. Sci Data. 10.1038/s41597-023-02303-y PMC10290071 37353567

[CR19] Calders K, Verbeeck H, Burt A et al (2022) Laser scanning reveals potential underestimation of biomass carbon in temperate forest. Ecol Solut Evid 3(4):e12197

[CR20] Cao B, Liu Q, Du Y et al (2019) A review of earth surface thermal radiation directionality observing and modeling: Historical development, current status and perspectives. Remote Sens Environ 232:111304

[CR21] CEOS-WGCV (2023) Roadmap towards an assessment framework for ceos-fiducial reference measurements (frms). Tech. rep., CEOS WGCV, URL https://calvalportal.ceos.org/documents/10136/958898/CEOS-FRM_Assessment_Framework_V1.pdf/a8318317-9f64-6f02-44db-e64f234c4036?t=1697787396736

[CR22] Chan K, Schillereff DN, Thompson J (2020) Low-cost electronic sensors for environmental research: pitfalls and opportunities. Prog Phys Geogr Earth Environ. 10.1177/0309133320956567

[CR23] Compernolle S, Verhoelst T, Pinardi G et al (2020) Validation of Aura-OMI QA4ECV climate data records with ground-based DOAS networks: the role of measurement and comparison uncertainties. Atmos Chem Phys 20(13):8017–8045. 10.5194/acp-20-8017-2020

[CR24] Da Silva E, Woolliams ER, Picot N et al (2023) Towards operational fiducial reference measurement (FRM) data for the calibration and validation of the sentinel-3 surface topography mission over inland waters, sea ice, and land ice. Remote Sens. 10.3390/rs15194826

[CR25] Divine DV, Dick C (2006) Historic variability of sea ice edge position in the Nordic seas. J Geophys Res 111(C1):C01001. 10.1029/2004JC002851

[CR26] Donlon CJ, Minnett PJ, Fox N et al (2014) Strategies for the laboratory and field deployment of ship-borne fiducial reference thermal infrared radiometers in support of satellite-derived sea surface temperature climate data records. In: Zibordi G, Donlon CJ, Parr AC (eds) Optical Radiometry for Ocean Climate Measurements, Experimental Methods in the Physical Sciences, vol 47. Academic Press, chap 5.2, pp 557–603, 10.1016/B978-0-12-417011-7.00018-0

[CR27] Dowling TP, Langsdale MF, Ermida SL et al (2022) A new east African satellite data validation station: performance of the LSA-SAF all-weather land surface temperature product over a savannah biome. ISPRS J Photogramm Remote Sens 187:240–258. 10.1016/j.isprsjprs.2022.03.003

[CR28] Duan SB, Li ZL, Li H et al (2019) Validation of collection 6 modis land surface temperature product using in situ measurements. Remote Sens Environ 225:16–29. 10.1016/j.rse.2019.02.020

[CR29] Duncanson L, Armston J, Disney M et al (2020) Global aboveground biomass product validation best practices protocol, version 1.0. Tech. rep., Land Product Validation Subgroup (WGCV/CEOS), 10.5067/doc/ceoswgcv/lpv/agb.001

[CR30] EDAP (2022) Earth observation mission quality assessment framework. edap.rep.001 issue 2.2. Tech. rep., EDAP, URL https://earth.esa.int/eogateway/activities/edap/edap-best-practice-guidelines

[CR31] Ermida SL, Trigo IF, DaCamara CC et al (2019) Quantifying the clear-sky bias of satellite land surface temperature using microwave-based estimates. J Geophys Res Atmos 124(2):844–857. 10.1029/2018JD029354

[CR32] EU Copernicus FRM4SOC-2 Project / EUMETSAT (2025) Copernicus frm4soc-2025 training in above-water radiometry. https://frm4soc2.eumetsat.int/post/copernicus-frm4soc-2025-training-above-water-radiometry, last accessed 4 July 2025

[CR33] Fioletov VE, Labow G, Evans R et al (2008) Performance of the ground-based total ozone network assessed using satellite data. J Geophys Res Atmos. 10.1029/2008JD009809

[CR34] Fons S, Kurtz N, Bagnardi M (2023) A decade-plus of antarctic sea ice thickness and volume estimates from cryosat-2 using a physical model and waveform fitting. Cryosphere 17(6):2487–2508. 10.5194/tc-17-2487-2023

[CR35] GCOS (2022) The 2022 GCOS implementation plan (GCOS-244). Tech. rep., World Meteorological Institution, URL https://library.wmo.int/records/item/58104-the-2022-gcos-implementation-plan-gcos-244

[CR36] Ghent D, Anand JS, Veal K et al (2024) The operational and climate land surface temperature products from the sea and land surface temperature radiometers on sentinel-3a and 3b. Remote Sens. 10.3390/rs16183403

[CR37] Goryl P, Fox N, Donlon C et al (2023) Fiducial reference measurements (frms): What are they? Remote Sens. 10.3390/rs15205017

[CR38] Grosvenor DP, Sourdeval O, Zuidema P et al (2018) Remote sensing of droplet number concentration in warm clouds: a review of the current state of knowledge and perspectives. Rev Geophys 56(2):409–453. 10.1029/2017RG000593 PMC6099364 30148283

[CR39] Gruber A, Su CH, Zwieback S et al (2016) Recent advances in (soil moisture) triple collocation analysis. Int J Appl Earth Observ Geoinf 45:200–211. 10.1016/j.jag.2015.09.002

[CR40] Guillevic P, Göttsche F, Nickeson J et al (2018) Land surface temperature product validation best practice protocol. version 1.1.0. Tech. rep., Land Product Validation Subgroup (WGCV/CEOS), 10.5067/doc/ceoswgcv/lpv/lst.001

[CR41] Göttsche FM, Olesen FS, Trigo IF et al (2016) Long term validation of land surface temperature retrieved from msg/seviri with continuous in-situ measurements in Africa. Remote Sens. 10.3390/rs8050410

[CR42] Häfner D, Gemmrich J, Jochum M (2018) Machine-guided discovery of a real-world rogue wave model. Proc Natl Acad Sci. 10.1073/pnas.2306275120 PMC10691345 37983488

[CR43] Hersbach H, Bell B, Berrisford P et al (2020) The era5 global reanalysis. Q J R Meteorol Soc 146(730):1999–2049. 10.1002/qj.3803

[CR44] Holben B, Eck T, Slutsker I et al (1998) Aeronet-a federated instrument network and data archive for aerosol characterization. Remote Sens Environ 66(1):1–16. 10.1016/S0034-4257(98)00031-5

[CR45] Hook SJ, Cawse-Nicholson K, Barsi J et al (2020) In-flight validation of the ecostress, landsats 7 and 8 thermal infrared spectral channels using the lake tahoe ca/nv and salton sea ca automated validation sites. IEEE Trans Geosci Remote Sens 58(2):1294–1302. 10.1109/TGRS.2019.2945701

[CR46] Hulley G, Shivers S, Wetherley E et al (2019) New ecostress and modis land surface temperature data reveal fine-scale heat vulnerability in cities: a case study for los angeles county, california. Remote Sens. 10.3390/rs11182136

[CR47] Hulley GC, Göttsche FM, Rivera G et al (2021) Validation and quality assessment of the ecostress level-2 land surface temperature and emissivity product. IEEE Trans Geosci Remote Sens. 10.1109/TGRS.2021.3079879

[CR48] Jiménez-Muñoz JC, Sobrino JA (2006) Error sources on the land surface temperature retrieved from thermal infrared single channel remote sensing data. Int J Remote Sens 27:999–1014. 10.1080/01431160500075907

[CR49] Kahn RA, Andrews E, Brock CA et al (2023) Reducing aerosol forcing uncertainty by combining models with satellite and within-the-atmosphere observations: a three-way street. Rev Geophys 61(2):e2022RG000796. 10.1029/2022RG000796

[CR50] Karagali I, Barfod Suhr M, Mottram R et al (2022) A new level 4 multi-sensor ice surface temperature product for the Greenland ice sheet. Cryosphere 16(9):3703–3721. 10.5194/tc-16-3703-2022

[CR51] Kennedy J, Smith R, Rayner N (2012) Using AATSR data to assess the quality of in situ sea-surface temperature observations for climate studies. Remote Sens Environ 116:79–92. 10.1016/j.rse.2010.11.021

[CR52] Kennedy JJ, Rayner NA, Smith RO et al (2011) Reassessing biases and other uncertainties in sea surface temperature observations measured in situ since 1850: 2. Biases and homogenization. J Geophys Res Atmos. 10.1029/2010JD015220

[CR53] Keppens A, Lambert JC, Granville J et al (2015) Round-robin evaluation of nadir ozone profile retrievals: methodology and application to metop-a gome-2. Atmos Meas Tech 8(5):2093–2120. 10.5194/amt-8-2093-2015

[CR54] Kern S, Lavergne T, Notz D et al (2019) Satellite passive microwave sea-ice concentration data set intercomparison: closed ice and ship-based observations. Cryosphere 13(12):3261–3307. 10.5194/tc-13-3261-2019

[CR55] Kern S, Lavergne T, Pedersen LT et al (2022) Satellite passive microwave sea-ice concentration data set intercomparison intercomparison using landsat data. Cryosphere 16(1):349–378. 10.5194/tc-16-349-2022

[CR56] Kharif C, Pelinovsky E (2003) Physical mechanisms of the rogue wave phenomenon. Eur J Mech B/Fluids 22:603–634. 10.1016/j.euromechflu.2003.09.002

[CR57] Kwok R (2018) Arctic sea ice thickness, volume, and multiyear ice coverage: losses and coupled variability (1958–2018). Environ Res Lett 13:105005. 10.1088/1748-9326/aae3ec

[CR58] Kwok R, Rothrock D (2009) Decline in arctic sea ice thickness from submarine and icesat records: 1958–2008. Geophys Res Lett 36(15)

[CR59] Lacagnina C, Doblas-Reyes F, Larnicol G et al (2022) Quality management framework for climate datasets. Data Sci J. 10.5334/dsj-2022-010

[CR60] Lambert JC, De Clercq C, von Clarmann T (2012) Ground-based remote sensing and in-situ methods for monitoring atmospheric water vapour - Chapter 9: Comparing and merging water vapour observations: a multi-dimensional perspective on smoothing and sampling issues, ISSI, issue 9, pp 177–199

[CR61] Lambert JC, Keppens A, Compernolle S et al (2024) Quarterly validation report of the copernicus sentinel-5 precursor operational data products issue 24: April 2018 august 2024. Tech. rep, ESA

[CR62] Langsdale MF, Dowling TPF, Wooster M et al (2020) Inter-comparison of field- and laboratory-derived surface emissivities of natural and manmade materials in support of land surface temperature (lst) remote sensing. Remote Sens 12:4127. 10.3390/rs12244127

[CR63] Langsdale MF, Wooster M, Harrison JJ et al (2021) Spectral emissivity (SE) measurement uncertainties across 2.5 14 um derived from a round-robin study made across international laboratories. Remote Sens. 10.3390/rs13010102

[CR64] Le Menn M, Poli P, David A et al (2019) Development of surface drifting buoys for fiducial reference measurements of sea-surface temperature. Front Mar Sci. 10.3389/fmars.2019.00578

[CR65] Li ZL, Tang BH, Wu H et al (2013) Satellite-derived land surface temperature: Current status and perspectives. Remote Sens Environ 131:14–37. 10.1016/j.rse.2012.12.008

[CR66] Loew A, Bell W, Brocca L et al (2017) Validation practices for satellite-based earth observation data across communities. Rev Geophys 55:779–817. 10.1002/2017RG000562

[CR67] Ma L, Zhao Y, Woolliams ER et al (2020) Uncertainty analysis for RadcalNet instrumented test sites using the Baotou sites BTCN and BSCN as examples. Remote Sens. 10.3390/rs12111696

[CR68] MacGregor JA, Boisvert LN, Medley B et al (2021) The scientific legacy of Nasa’s operation Icebridge

[CR69] Malakar N, Hulley GC, Hook SJ et al (2018) An operational land surface temperature product for LANDSAT thermal data: methodology and validation. IEEE Trans Geosci Remote Sens 56(10):5717–5735. 10.1109/TGRS.2018.2824828

[CR70] Martin MA, Ghent D, Pires AC et al (2019) Comprehensive in situ validation of five satellite land surface temperature data sets over multiple stations and years. Remote Sens. 10.3390/rs11050479

[CR71] McGarragh GR, Poulsen CA, Thomas GE et al (2018) The community cloud retrieval for climate (CC4CL) - Part 2: the optimal estimation approach. Atmos Meas Tech 11(6):3397–3431. 10.5194/amt-11-3397-2018

[CR72] Meerdink SK, Hook SJ, Roberts DA et al (2019) The ecostress spectral library version 10. Remote Sens Environ. 10.1016/j.rse.2019.05.015

[CR73] Merchant CJ, Paul F, Popp T et al (2017) Uncertainty information in climate data records from earth observation. Earth Syst Sci Data 9(2):511–527. 10.5194/essd-9-511-2017

[CR74] Michel A, Granero-Belinchon C, Cassante C et al (2021) A new material-oriented TES for land surface temperature and suhi retrieval in urban areas: case study over Madrid in the framework of the future Trishna mission. Remote Sens. 10.3390/rs13245139

[CR75] Millington JDA, Perkins O, Smith C (2022) Human fire use and management: a global database of anthropogenic fire impacts for modelling. Fire. 10.3390/fire5040087

[CR76] Mittaz J, Merchant CJ, Woolliams ER (2019) Applying principles of metrology to historical earth observations from satellites. Metrologia 56(3):032002

[CR77] Niclòs R, Puchades J, Coll C et al (2021) Evaluation of LANDSAT-8 TIRS data recalibrations and land surface temperature split-window algorithms over a homogeneous crop area with different phenological land covers. ISPRS J Photogramm Remote Sens 174:237–253. 10.1016/j.isprsjprs.2021.02.005

[CR78] Niro F, Goryl P, Dransfeld S et al (2021) European space agency (ESA) calibration/validation strategy for optical land-imaging satellites and pathway towards interoperability. Remote Sens 13(15):3003

[CR79] NPL (2024) Comet toolkit. URL https://www.comet-toolkit.org/, Accessed on Nov 13, 2024

[CR80] O’Carroll A, Armstrong E, Beggs H et al (2019) Observational needs of sea surface temperature. Front Mar Sci. 10.3389/fmars.2019.00420

[CR81] Pan X, Wang Z, Liu S et al (2024) The impact of clear-sky biases of land surface temperature on monthly evapotranspiration estimation. Int J Appl Earth Obs Geoinf 129:103811. 10.1016/j.jag.2024.103811

[CR82] Popp T, Mittaz J (2022) Systematic propagation of AVHRR AOD uncertainties–a case study to demonstrate the FIDUCEO approach. Remote Sens. 10.3390/rs14040875

[CR83] Povey AC, Grainger RG (2015) Known and unknown unknowns: uncertainty estimation in satellite remote sensing. Atmos Meas Tech 8(11):4699–4718. 10.5194/amt-8-4699-2015

[CR84] Ramo R, Roteta E, Bistinas I et al (2021) African burned area and fire carbon emissions are strongly impacted by small fires undetected by coarse resolution satellite data. Proc Natl Acad Sci. 10.1073/pnas.2011160118 PMC7936338 33619088

[CR85] Remer L, Kaufman Y, Tanre D et al (2005) The Modis aerosol algorithm, products, and validation. J Atmos Sci 62:947–973

[CR86] Rodgers CD, Connor BJ (2003) Intercomparison of remote sounding instruments. J Geophys Res 108(D3):4116. 10.1029/2002JD002299

[CR87] Rostosky P, Spreen G, Farrell SL et al (2018) Snow depth retrieval on arctic sea ice from passive microwave radiometers–improvements and extensions to multiyear ice using lower frequencies. J Geophys Res Oceans 123:7120–7138. 10.1029/2018JC014028

[CR88] Rothrock DA, Wensnahan M (2007) The accuracy of sea ice drafts measured from U.S. navy submarines. J Atmos Oceanic Tech 24(11):1936–1949. 10.1175/JTECH2097.1

[CR89] Ru C, Duan SB, Jiang XG et al (2023) An extended SW-TES algorithm for land surface temperature and emissivity retrieval from Ecostress thermal infrared data over urban areas. Remote Sens Environ 290:113544. 10.1016/j.rse.2023.113544

[CR90] Ruddick KG, Brando VE, Corizzi A et al (2024) Waterhypernet: a prototype network of automated in situ measurements of hyperspectral water reflectance for satellite validation and water quality monitoring. Front Remote Sens. 10.3389/frsen.2024.1347520

[CR91] Sayer AM, Govaerts Y, Kolmonen P et al (2020) A review and framework for the evaluation of pixel-level uncertainty estimates in satellite aerosol remote sensing. Atmos Meas Tech 13(2):373–404. 10.5194/amt-13-373-2020

[CR92] Schutgens N, Partridge DG, Stier P (2016) The importance of temporal collocation for the evaluation of aerosol models with observations. Atmos Chem Phys 16:1065–1079. 10.5194/acp-16-1065-2016

[CR93] Schutgens N, Sayer AM, Heckel A et al (2020) An aerocom aerosat study: intercomparison of satellite AOD datasets for aerosol model evaluation. Atmos Chem Phys 20:12431–12457. 10.5194/acp-20-12431-2020

[CR94] Schutgens NAJ (2020) Site representativity of AERONET and GAW remotely sensed aerosol optical thickness and absorbing aerosol optical thickness observations. Atmos Chem Phys 20(12):7473–7488. 10.5194/acp-20-7473-2020

[CR95] Schutgens NAJ, Gryspeerdt E, Weigum N et al (2016) Will a perfect model agree with perfect observations? the impact of spatial sampling. Atmos Chem Phys 16:6335–6353. 10.5194/acp-16-6335-2016

[CR96] Sha MK, De Mazière M, Notholt J et al (2024) Fiducial reference measurement for greenhouse gases (FRM4GHG). Remote Sens. 10.3390/rs16183525

[CR97] Sparling LC, Wei JC, Avallone LM (2006) Estimating the impact of small-scale variability in satellite measurement validation. J Geophys Res Atmos. 10.1029/2005JD006943

[CR98] Su Z, Timmermans W, Zeng Y et al (2018) An overview of European efforts in generating climate data records. Bull Am Meteor Soc 99(2):349–359. 10.1175/BAMS-D-16-0074.1

[CR99] Sánchez-Zapero J, Camacho F, Martínez-Sánchez E et al (2023) Global estimates of surface albedo from sentinel-3 OLCI and SLSTR data for COPERNICUS climate change service: Algorithm and preliminary validation. Remote Sens Environ 287:113460. 10.1016/j.rse.2023.113460

[CR100] Teutsch I (2022) Rogue waves in the southern north sea. PhD thesis, Staats-und Universitätsbibliothek Hamburg Carl von Ossietzky

[CR101] Teutsch I, Weisse R, Moeller J et al (2020) A statistical analysis of rogue waves in the southern north sea. Nat Hazard 20:2665–2680. 10.5194/nhess-20-2665-2020

[CR102] Thorne P, Schulz J, Tan D et al (2015) Gap analysis for integrated atmospheric ECV climate monitoring: Report on system of systems approach adopted and rationale, GAIA-CLIM measurement maturity matrix guidance. deliverable 1.3. Tech. rep., Horizon 2020 GAIA-CLIM project

[CR103] Van Roozendael M, Hendrick F, Friedrich MM et al (2024) Fiducial reference measurements for air quality monitoring using ground-based max-DOAS instruments (FRM4DOAS). Remote Sens. 10.3390/rs16234523

[CR104] Vandaele A, Hermans C, Simon P et al (1998) Measurements of the NO2 absorption cross-section from 42000 cm-1 to 10000 cm-1 (238–1000 nm) at 220 K and 294K. J Quant Spectrosc Radiat Transf 59:171–184. 10.1016/s0022-4073(97)00168-4

[CR105] Verhoelst T, Granville J, Hendrick F et al (2015) Metrology of ground-based satellite validation: co-location mismatch and smoothing issues of total ozone comparisons. Atmos Meas Tech 8(12):5039–5062. 10.5194/amt-8-5039-2015

[CR106] Verhoelst T, Compernolle S, Pinardi G et al (2021) Ground-based validation of the Copernicus sentinel-5p Tropomi measurements with the NDACC ZSL-DOAS, max-DOAS and Pandonia global networks. Atmos Meas Tech 14(1):481–510. 10.5194/amt-14-481-2021

[CR107] Verhoelst T, Povey AC, Gruber A et al (2025) Confidently uncertain: Validating satellite ECV uncertainty estimates. Surv Geophys

[CR108] Vigouroux C, Langerock B, Bauer Aquino CA et al (2020) TROPOMI-Sentinel-5 Precursor formaldehyde validation using an extensive network of ground-based Fourier-transform infrared stations. Atmos Meas Tech 13(7):3751–3767. 10.5194/amt-13-3751-2020

[CR109] Virtanen TH, Kolmonen P, Sogacheva L et al (2018) Collocation mismatch uncertainties in satellite aerosol retrieval validation. Atmos Meas Tech 11:925–938. 10.5194/amt-11-925-2018

[CR110] Wensnahan M, Rothrock DA (2005) Sea-ice draft from submarine-based sonar: Establishing a consistent record from analog and digitally recorded data. Geophys Res Lett 32(11):L11502. 10.1029/2005GL022507

[CR111] Wilkinson MD, Dumontier M, Aalbersberg IJ et al (2016) The fair guiding principles for scientific data management and stewardship. Sci Data. 10.1038/sdata.2016.18 PMC4792175 26978244

[CR112] Wimmer W, Robinson IS (2016) The ISAR instrument uncertainty model. J Atmos Oceanic Tech 33(11):2415–2433. 10.1175/JTECH-D-16-0096.1

[CR113] Wooster MJ, Langsdale MF, Hook SJ et al (In Review) Inter-comparison and in-situ validation of land surface temperature and emissivity retrieved over European landscapes from the hytes and tasi airborne LWIR hyperspectral imagers and ecostress on the ISS, in Review

[CR114] Worby AP, Comiso JC (2004) Studies of the Antarctic sea ice edge and ice extent from satellite and ship observations. Remote Sens Environ 92(1):98–111. 10.1016/j.rse.2004.05.007

[CR115] Yamamoto H, Irie H (2023) Validation of himawari-8 ahi level-2 and level-3 aerosol products with modis and ground-based data under clear-sky conditions. In: IGARSS 2023 - 2023 IEEE international geoscience and remote sensing symposium, pp 4498–4501, 10.1109/IGARSS52108.2023.10281930

[CR116] Zhang B, Liu J, Wang Z et al (2020) Antarctic ice-shelf thickness changes from CRYOSAT-2 sarin mode measurements: assessment and comparison with icebridge and icesat. J Earth Syst Sci 129:1–15

[CR117] Zhou L, Xu S, Liu J et al (2018) On the retrieval of sea ice thickness and snow depth using concurrent laser altimetry and l-band remote sensing data. Cryosphere 12(3):993–1012. 10.5194/tc-12-993-2018

[CR118] Zhou L, Stroeve J, Xu S et al (2021) Inter-comparison of snow depth over arctic sea ice from reanalysis reconstructions and satellite retrieval. Cryosphere 15(1):345–367. 10.5194/tc-15-345-2021

[CR119] Zibordi C, Donlon CJ, Parr AC (2014) Optical radiometry for ocean climate measurements. Experimental methods in the physical sciences. Academic Press

